# Extremely rapid and reversible optogenetic perturbation of nuclear proteins in living embryos

**DOI:** 10.1016/j.devcel.2021.07.011

**Published:** 2021-08-23

**Authors:** Anna C. Kögler, Yacine Kherdjemil, Katharina Bender, Adam Rabinowitz, Raquel Marco-Ferreres, Eileen E.M. Furlong

**Affiliations:** 1 European Molecular Biology Laboratory (EMBL), Genome Biology Unit, Heidelberg 69117, Germany

**Keywords:** rapid protein depletion, conditional loss-of-function, embryogenesis, optogenetics, iLEXY, nuclear proteins, transcription factor, Twist, gene expression, reversible perturbation

## Abstract

Many developmental regulators have complex and context-specific roles in different tissues and stages, making the dissection of their function extremely challenging. As regulatory processes often occur within minutes, perturbation methods that match these dynamics are needed. Here, we present the improved light-inducible nuclear export system (iLEXY), an optogenetic loss-of-function approach that triggers translocation of proteins from the nucleus to the cytoplasm. By introducing a series of mutations, we substantially increased LEXY’s efficiency and generated variants with different recovery times. iLEXY enables rapid (t_1/2_ < 30 s), efficient, and reversible nuclear protein depletion in embryos, and is generalizable to proteins of diverse sizes and functions. Applying iLEXY to the *Drosophila* master regulator Twist, we phenocopy loss-of-function mutants, precisely map the Twist-sensitive embryonic stages, and investigate the effects of timed Twist depletions. Our results demonstrate the power of iLEXY to dissect the function of pleiotropic factors during embryogenesis with unprecedented temporal precision.

## Highlights

• Improved optogenetic nuclear protein depletion gives efficient perturbation *in vivo*
• iLEXY depletes proteins from the nucleus in seconds, with tunable recovery kinetics
• iLEXY is generalizable to diverse proteins and compatible with many assays
• Nuclear Twist depletion phenocopies loss-of-function mutants in *Drosophila* embryos

## Graphical abstract

**Figure:**
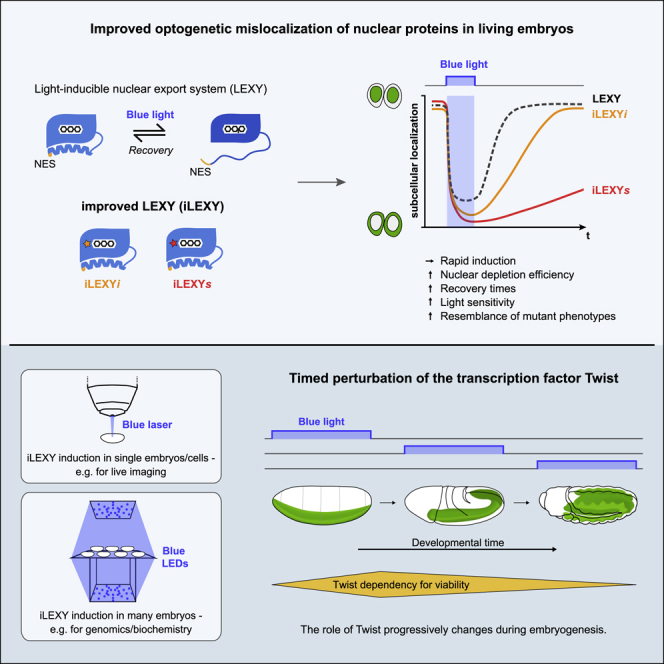

## Teaser

Kögler et al. establish an improved optogenetic tool (iLEXY) for conditional, efficient perturbation of nuclear proteins in embryos. iLEXY can mimic loss-of-function mutants and interrogate the function of essential, pleiotropic proteins. Using iLEXY, Kögler et al. uncover the temporal requirement and function of the *Drosophila* regulator Twist at unprecedented resolution.

## Introduction

Many proteins that govern key biological processes are pleiotropic in function. For example, the same transcription factor (TF), or signaling pathway component, can regulate different processes in different cell types or in the same cell type at different time points or conditions (Carroll et al., 2013). When these proteins are essential, dissecting their context-dependent function is extremely challenging, especially during embryogenesis where every step builds on the last. Genetic loss-of-function approaches often have severe phenotypes, complicating the investigation of the protein’s role after its first essential function due to the accumulation of direct and indirect effects. Conditional genetic knockouts or knockdowns, for example, CRE/loxP-based systems (McLellan et al., 2017) or shRNA expression (Enerly et al., 2002; Szulc et al., 2006), address some of these issues, allowing a gene to be targeted in a specific tissue and stage. However, their dynamics are limited by the turnover of the encoded protein (often in the range of hours or days Milo and Phillips, 2015), and knockouts are irreversible by nature. Degradation-based approaches can function within 10 min–1 h (Clift et al., 2017; Irizarry et al., 2020) but are slow in their reversibility. These systems are, therefore, too slow to functionally dissect highly dynamic processes occurring within seconds to minutes, including many steps in gene regulation and signal transduction. Alternative perturbation approaches include the direct modulation of a protein’s function or its subcellular localization, with the latter being easier to generalize for diverse proteins (Gautier et al., 2014).

Transferring such approaches to intact living embryos remains a challenge. Methods such as the anchor-away system (Haruki et al., 2008) induced by chemicals or antibodies have limited application in multicellular systems as the delivery of the drug is often slow, inefficient, and uneven. An alternative stimulus, which enters samples with high precision, is light. This has led to growing interest in optogenetics — genetically encoded systems that couple light-based stimulation with changes in protein activity (reviewed in Johnson and Toettcher (2018); Khamo et al. (2017); Kowalik and Chen (2017); Krueger et al. 2019). Despite these advances, loss-of-function optogenetic approaches for nuclear proteins in embryos are sparse. The blue-light-sensitive CRY2 domain has recently been applied to the *Drosophila* TF Bicoid (Huang et al., 2017). A light-induced conformational change of the CRY2 domain could interfere with the fused Bicoid protein’s function. However, the outcome of CRY2 fusions is difficult to predict as its functional effect (whether it creates a gain- or loss-of-function), its mechanism of perturbation, and its reversibility vary depending on the protein (Bugaj et al., 2013; Huang et al., 2017; Kaur et al., 2017; Viswanathan et al., 2019). In the case of Bicoid, the exact mechanism of perturbation remained unclear (Huang et al., 2017). Both the spatial distribution of the TF and its DNA-binding were unchanged, making the direct quantification of the perturbation’s efficiency and dynamics impossible.

To obtain precise temporal control of nuclear protein function in embryos, we set out to establish a generalizable, efficient, and highly dynamic optogenetic system. We explored the potential of LEXY (light-inducible nuclear export system) to function in embryos, a blue-light-inducible system shown to translocate nuclear proteins to the cytoplasm in mammalian cell culture (Niopek et al., 2016). Through a series of mutations, we considerably increased LEXY’s nuclear depletion efficiency and tuned the system to different light sensitivities and recovery times. We transferred the resulting improved LEXY (iLEXY) variants to the *Drosophila* embryo and showed that the system enables extremely rapid (t_1/2_ < 30 s), reversible (t_1/2_ ∼5–50 min depending on the variant), and efficient nuclear depletion.

By fusion to the endogenous gene, we applied iLEXY to the well-studied TF Twist, a master regulator of mesoderm development. Twist is first required for the gastrulation of the presumptive mesoderm (Nüsslein-Volhard et al., 1984; Simpson, 1983) and subsequently for its development into different muscle primordia (Baylies and Bate, 1996; Wong et al., 2014). During these developmental stages, Twist binds to different sets of enhancers, often together with stage-specific TFs (Sandmann et al., 2007; Wilczyński and Furlong, 2010; Yáñez-Cuna et al., 2012; Zinzen et al., 2009). Despite decades of research, a number of open questions remain, especially with regard to Twist’s function post-gastrulation. In *twist* loss-of-function mutants, these later processes are masked by early gastrulation defects, which result in the absence of any mesoderm-derived muscle lineages. Here, we demonstrate that iLEXY-mediated nuclear depletion of Twist phenocopies the genetic loss-of-function allele and results in the loss of known target genes’ expression, confirming the successful perturbation of the TF’s function. We then utilize iLEXY to precisely map the Twist-sensitive developmental time window and to investigate the effects of timed Twist depletions. Our results reveal a progressive change in the role of Twist and highlight the power of iLEXY to temporally disentangle the function of pleiotropic regulators. The strategy presented here can be applied to many nuclear proteins, with diverse molecular functions, in *Drosophila* and likely many other model systems.

## Results

### iLEXY enables light-inducible, rapid, reversible, and efficient mislocalization of nuclear proteins in living embryos

**Figure 1:**
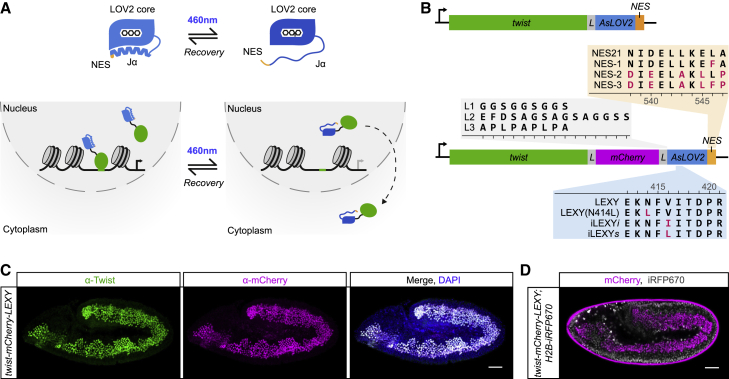
Development of iLEXY in embryos using endogenously tagged *twist* (A) The optogenetic nuclear export system LEXY. The modified Jα helix of the *As*LOV2 domain (blue) contains a NES (orange), which becomes accessible to the nuclear export machinery upon blue light exposure (460 nm), allowing light-inducible nuclear depletion of LEXY-fused proteins (green). In the dark, the NES becomes buried again in the Jα helix. (B) LEXY cassettes, with and without mCherry, fused to the endogenous *twist* coding sequence. LEXY variants were created by mutating the LOV2 domain (blue), the NES (orange), and by substituting the linker (L) region (gray). Residue numbering is based on full-length *As*LOV2. (C and D) Expression and subcellular localization of Twist-mCherry-LEXY in homozygous, dark-incubated embryos visualized by immunostaining against Twist and mCherry (C) and by live imaging of mCherry (D). Nuclei were stained with DAPI (C) or marked by Histone H2B-fused iRFP670 (D). Dorsal up, anterior left; scale bars, 50 μm. See also Figure S1.

LEXY, initially developed by Niopek et al. in mammalian cell lines (Niopek et al., 2016), is composed of a blue-light-sensitive *As*LOV2 domain (Christie et al., 1999; Wu et al., 2009), which contains a synthetic nuclear export sequence (NES) embedded in its C-terminal Jα helix (Figure 1A). In the dark state, the NES is buried within the Jα helix and, therefore, inaccessible to the CRM1 nuclear export machinery. However, upon blue light excitation of the LOV2 domain (∼460 nm), the helix partially unwinds, dissociates from the core domain, and exposes the NES. This conformational change is fully reversed following the removal of blue light. Fused to a protein of interest, the system can thereby allow light to control the protein’s nuclear export (Figure 1A).

Here, we set out to make a number of improvements to the system to perturb nuclear protein function in embryos: first, to increase the mislocalization efficiency to sufficiently perturb the molecular and developmental processes of interest *in vivo*. Second, to create LEXY variants with different recovery times, facilitating the dissection of processes occurring at various time scales. Third, to facilitate the use of the optogenetic system with a wide range of proteins. And fourth, to transport the system to embryos and optimize its activation conditions in the *in vivo* situation. To enable this, we started from the initial LEXY cassette (Niopek et al., 2016) and created eight different variations, targeting the LOV2 domain, the NES, and the linker region (Figure 1B). We tested the impact of three mutation combinations in the NES to potentially increase its strength and two new linker regions between mCherry and the LOV2 domain: a long flexible linker (L2) and a rigid proline linker (L3) preferred by some proteins. Moreover, we generated three individual point mutations in the LOV2 domain (N414L, V416I, or V416L). These mutations increase the half-time of the LOV2 domain’s dark recovery *in vitro* and are, therefore, called slow-cycling variants (Kawano et al., 2013; Zayner and Sosnick, 2014; Zoltowski et al., 2009). We reasoned that they could increase LEXY’s recovery time, as a consequence, shift the equilibrium between the Jα helix folded and unfolded state further toward the latter (Diensthuber et al., 2014), and thereby help to improve LEXY’s nuclear depletion efficiency.

To examine the ability of each variant to mislocalize nuclear proteins, we performed (1) a quick qualitative yes/no assessment in transiently transfected *Drosophila* cell culture and (2) a detailed quantitative assessment of its kinetics and nuclear depletion efficiency in embryos using endogenously tagged proteins. Tissue culture assays revealed that mutations to the NES and the substitution of the short flexible linker for a longer one had no obvious improvement to the efficiency of nuclear depletion for the tested proteins. The proline linker, on the other hand, prevented a successful mislocalization. The most promising results were obtained for the slow-cycling LOV2 variants, hereafter called improved LEXY (iLEXY).

To determine the power of iLEXY in embryos, we selected the master regulator of *Drosophila* mesoderm development, Twist. Using CRISPR-Cas9-mediated homology-directed repair, we generated five knockin lines, tagging the endogenous *twist* gene at the C terminus with the initial LEXY construct with or without mCherry and with the three slow-cycling iLEXY variants with mCherry (Figure 1B). Importantly, all resulting *Drosophila* lines, except for the variant N414L, are homozygous viable and fertile when kept under safelight conditions (hereafter referred to as “dark”). Moreover, the fusion proteins of Twist with LEXY and its variants V416I and V416L faithfully recapitulate the wild-type Twist spatiotemporal expression and localize to the nucleus in the dark (Figures 1C, S1A, and S1B). This indicates that the presence of these tags does not impair Twist function, expression, or subcellular localization in the absence of blue light. The N414L variant, which is homozygous lethal, is detected primarily in the cytoplasm (Figure S1C) even under safelight conditions, suggesting that this LOV2 mutation creates a Jα helix with constitutively exposed NES. This variant was not pursued further.

**Figure 2:**
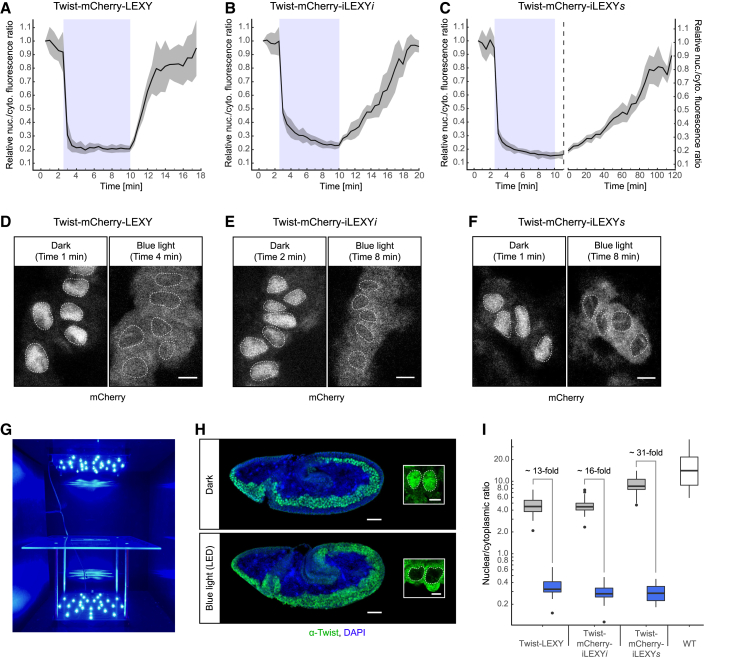
iLEXY enables rapid efficient nuclear protein export with tunable recovery times in embryos (A–F) Blue-light-induced changes in Twist-mCherry-LEXY (A and D), Twist-mCherry-iLEXY*i* (B and E), and Twist-mCherry-iLEXY*s* (C and F) localization observed by live confocal imaging of mCherry in stage 9–10 embryos. mCherry-expressing cells were imaged over a time course of blue light induction (blue rectangle, A–C) and subsequent recovery in the dark. (A–C) Mean (line) ± SD (shading) of the nuclear/cytoplasmic mCherry fluorescence ratio relative to the first time point. Note, for Twist-mCherry-iLEXY*s* (C) induction and recovery were recorded separately. See also Videos S1 and S2 and Figure S2. (D–F) Examples of the mCherry signal before (left panel) and during (right panel) blue light induction from time courses shown earlier. Dashed lines indicate the position of nuclei. Scale bars, 5 μm. (G–I) LED-based induction of iLEXY. (G) Inside the LED blue light box. The transparent sample stage is approximately equidistant from the LEDs at the top and bottom. (H) Subcellular localization of Twist-mCherry-iLEXY*s* visualized by α-Twist staining with DAPI in homozygous embryos incubated in the dark or 60 min under blue light in the LED box. Blue light induction (bottom) leads to nuclear Twist depletion and gastrulation defects. Dorsal up, anterior left; scale bars 50 μm. Insets show individual cells from the same embryos at high magnification. Nuclei are outlined by dashed lines. Scale bars, 5 μm. (I) Twist localization quantified as nuclear/cytoplasmic fluorescence ratios of α-Twist signal from cells of wild-type (WT) embryos (white boxplot) or of *twist-LEXY*, *twist-mCherry-iLEXYi*, and *twi-mCherry-iLEXYs* embryos incubated in the dark (gray) or LED blue light (blue boxplots) averaged across stages 6, 8, and 10. Figure S2F shows ratios for each embryonic stage separately. Examples of corresponding stains are shown in Figures 2H and S2E.

To measure the kinetics and nuclear depletion efficiency *in vivo*, we imaged the Twist-mCherry-LEXY and -iLEXY fusion proteins live in embryos expressing the nuclear fluorescent marker H2B-iRFP670. Importantly, the wavelengths used for the excitation of the red fluorophores do not induce an unfolding of the Jα helix and the system was only activated using a 458-nm laser. Quantification of the mCherry signal in the nucleus and cytoplasm indicate that all fusion proteins rapidly mislocalized from the nucleus to the cytoplasm with half-times of 30 s or less following blue light stimulation (Figures 2A–2F and S2A–S2D; Video S1). This change in localization was fully reversed in the dark, allowing the system to undergo multiple consecutive rounds of activation and recovery (Figure S2B). Full recovery was observed after ∼4 min for the initial LEXY construct and ∼10 and ∼120 min for the V416I and V416L variants, respectively (Figures 2A–2C; Video S2). Hereafter, we call these variants iLEXY*i* (iLEXY *intermediate*) and iLEXY*s* (iLEXY *slow*). The highest nuclear depletion efficiency was observed with iLEXY*s* (Figures 2C and 2F). These results show that iLEXY enables extremely rapid, reversible, and repeated mislocalization of an endogenous nuclear factor (Twist) to the cytoplasm in living embryos with kinetics that cannot be achieved by degradation- or expression-based systems. Moreover, LOV2 variants can tune iLEXY’s recovery kinetics and improve the system’s efficiency (Figure S2G).

Video S1. iLEXY enables extremely rapid nuclear protein export, related to Figures 2C and 2F Time-lapse movie showing the localization of Twist-mCherry-iLEXY*s* in a group of cells in a stage 10 embryo before and during blue light stimulation. The time (format minutes:seconds) and the blue light status (Blue light OFF and ON) are shown as an overlay on the movie. The quantification of Twist localization from this movie is included in Figures 2C and S2D (left panel). Note that the co-expressed nuclear marker (H2B-iRFP670) is not shown in this movie.

Video S2. iLEXY-mediated nuclear translocation is reversed in the absence of blue light, related to Figure 2C Time-lapse movie showing the localization of Twist-mCherry-iLEXY*s* in cells of the posterior half of a stage 11–12 embryo before and during dark recovery. The time (format hours:minutes:seconds) and the blue light status (Blue light ON and OFF) are shown as an overlay on the movie. The quantification of Twist localization from this movie is included in Figures 2C and S2D (right panel). Note that the co-expressed nuclear marker (H2B-iRFP670) is not shown in this movie.

Although induction of the optogenetic system with a microscope’s laser is ideal for live imaging-based experiments, immunostaining, genomics- and biochemistry-based experiments typically require more material. To simultaneously induce iLEXY in many embryos, we designed a programmable LED-based blue light illumination box. In this blue light box, samples are placed on a transparent stage, where they are exposed to blue light of the desired intensity and pulse-frequency from two directions (Figure 2G). Embryos incubated and formaldehyde-fixed under these conditions showed dramatic nuclear depletion of Twist in all embryos investigated (80/80) (Figures 2H and S2E). These fixed embryos also allowed for better quantification of the nuclear depletion compared with live imaging (see STAR Methods). When compared with dark-incubated embryos, a 13-, 16-, and 31-fold reduction in Twist nuclear/cytoplasmic ratio was observed for the original LEXY, iLEXY*i*, and iLEXY*s*, respectively (Figure 2I). The efficiency of nuclear depletion was very similar at different Twist-expressing developmental stages, including later stages (stages 8 and 10) when the target cells are internal, requiring blue light to penetrate deeper into the embryo (Figure S2F). This demonstrates that blue light LEDs can efficiently and reliably induce iLEXY-mediated nuclear protein depletion in many embryos in bulk and across different embryonic stages.

Finally, we examined the generalizability of iLEXY, focusing on iLEXY*s*, by testing its ability to mediate nuclear export of diverse proteins. The mislocalization of each protein was tested in a transient transfection-based assay in *Drosophila* tissue culture cells (Figure S3A). Of the 12 nuclear proteins tested, 5 proteins appeared unresponsive to blue light. These proteins either remained nuclear (e.g., *fs(1)h* in Figure S3A) or were already localized in the cytoplasm even in the dark (e.g., *prg*, Figure S3A). The latter scenario may be due to the Jα helix being trapped in an unfolded state, making the NES constitutively accessible. Nevertheless, the majority of proteins (∼60% [7/12]) very rapidly mislocalized to the cytoplasm in a blue-light-dependent manner. These successfully depleted nuclear proteins have diverse sizes and functions, including TFs, co-factors, and chromatin regulators (Figure S3B). The success rate was particularly high for direct DNA-binders such as TFs (∼80% [5/6]) and appeared lowest for components of multi-subunit complexes, perhaps because of the proteins high chromatin avidity or because the Jα helix is buried within the complex and unable to unfold.

In summary, iLEXY is an extremely rapid reversible system to manipulate the localization of many nuclear proteins, opening the door to address a wide range of questions about the proteins’ function in ways that were not possible before.

### iLEXY-mediated nuclear export of Twist recapitulates *twist* loss-of-function phenotypes

To determine whether Twist-iLEXY nuclear depletion is sufficient to recapitulate the *twist* loss-of-function mutant phenotype, we examined embryos homozygous for each of the LEXY- and iLEXY-tagged *twist* alleles in the dark and upon blue light exposure and compared them to embryos homozygous for the characterized *twist*^*1*^ loss-of-function (null) allele. Phenotypes of *twist*^*1*^ embryos include embryonic lethality, gastrulation defects, and the loss of mesoderm-derived muscles tissues.

**Figure 3:**
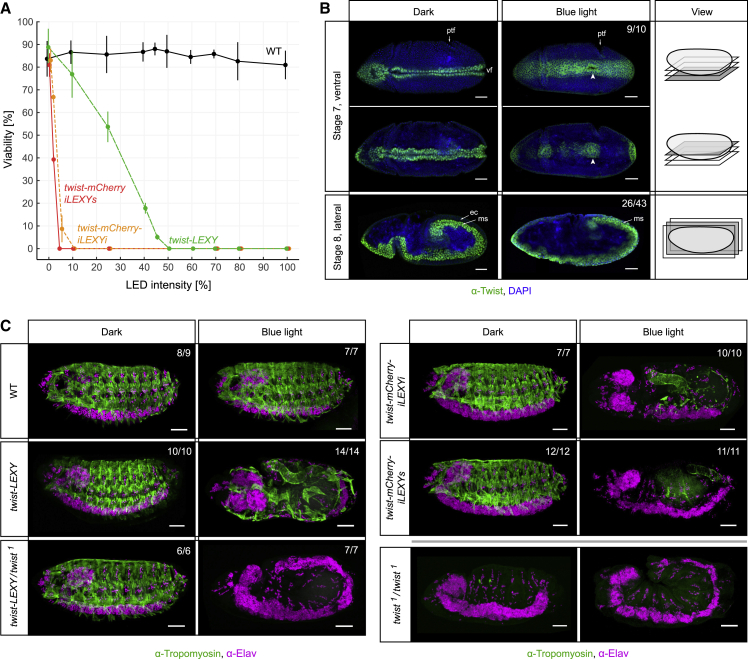
iLEXY-mediated nuclear Twist depletion phenocopies *twist* loss-of-function mutant (A) Blue-light-dependent viability of wild-type (WT) and homozygous embryos with *twist* tagged to different LEXY variants. Hatching assays of embryos to first instar wandering larvae were performed under increasing blue light LED intensities. Data points represent mean ± SD of at least two independent experiments and ∼50 embryos per experiment. (B) Gastrulation of ventral furrow (vf) cells and germ layer separation in homozygous *twist-mCherry-iLEXYs* embryos incubated continuously in the dark or blue light. Immunostaining with α-Twist antibody is shown as single plane ventral views of external and internal cells (stage 7 embryos) and as maximum intensity projections of lateral views (stage 8 embryos). Positions of the ectodermal (ec) and mesodermal (ms) layer are indicated by arrows. The presence of the posterior transverse furrow (ptf) was used to identify stage 7 embryos. Anterior left, dorsal up in bottom panel; scale bars, 50 μm. (C) Formation of mesoderm-derived muscles in nuclear Twist-depleted embryos. Z-projections show stage 16 embryos of the indicated genotypes incubated in the dark or blue light throughout embryogenesis. Wild-type (WT) and *twist* loss-of-function mutant embryos (*twist*^1^/*twist*^1^) are shown for comparison. α-tropomyosin and α-Elav immunostainings visualize muscles and nervous system, respectively. Dorsal up, anterior left; scale bars, 50 μm. The number of embryos with the presented phenotype is indicated (top right) in (B) and (C). See also Figure S4.

First, we measured the viability of embryos by performing hatching assays in the presence of different LED intensities. Embryos were placed under blue light in the LED box before reaching the first Twist-expressing stage (i.e., before stage 5). Wild-type (WT) embryos (without any insertion) showed a viability of 80%–90% regardless of the blue light intensity (Figures 3A and S4A), and hatched larvae developed into viable fertile adults at normal rates (Figure S4B). This indicates that blue light itself, at the intensities applied here, has no major effect on *Drosophila* embryogenesis. Similarly, when kept in the dark, 80%–90% of embryos of all *twist-LEXY-* and -*iLEXY*-tagged fly lines hatched at the end of embryogenesis (Figure 3A), demonstrating that the tag itself has no impact on viability. In contrast, with increasing blue light intensities, *twist*-*LEXY* and -*iLEXY* embryos failed to hatch (Figure 3A). The sensitivity of embryos to blue light correlated with the recovery time of the respective iLEXY variant: embryos carrying the slow (t_1/2_ ∼ 50 min) and intermediate variant (t_1/2_ ∼ 5 min) die at lower LED intensities than embryos carrying the initial LEXY construct (t_1/2_ ∼ 1.5 min) (Figure 3A). LEXY’s light sensitivity was also influenced by the presence of the mCherry fluorophore, although to a much lesser extent (Figure S4A). Blue-light-exposed embryos heterozygous for *twist-mCherry-iLEXYs* were not impaired in their survival (Figure S4C) and hatched larvae were morphologically indistinguishable from WT larvae. This suggests that the phenotype observed in homozygous embryos reflects the loss of Twist function in the nucleus rather than a gain of a potential unknown or neo-function in the cytoplasm, although subtle effects of the latter cannot be excluded. Overall, this demonstrates that optogenetic depletion of Twist from the nucleus is embryonic lethal, consistent with *twist* loss-of-function mutants, and indicates that iLEXY successfully perturbs the function of the TF.

Next, we examined if the morphogenetic defects characteristic of *twist*^*1*^ mutant embryos are observed upon iLEXY nuclear Twist depletion. During gastrulation, WT and iLEXY embryos incubated in the dark form a ventral furrow and internalize Twist-expressing cells of the presumptive mesoderm (Figures 3B, top left panel and S4D). As a result, an internal mesodermal and external ectodermal sheet of cells are formed (Figure 3B, bottom left panel [stage 8]). In *twist*^*1*^ mutant embryos, the ventral furrow is smaller and forms significantly later, and cells of the presumptive mesoderm are not internalized (Leptin and Grunewald, 1990; Wong et al., 2014; Figure S4D). *twist-mCherry-iLEXYs* embryos exposed to blue light starting from 45 min after egg lay (using the LED box) displayed very similar gastrulation defects. Cells, which would normally have nuclear Twist, formed a defective ventral furrow (9/10 embryos) and often failed to invaginate even during the subsequent developmental stages (26/43 embryos) (Figures 3B, right panel and S4D). These phenotypes were observed for the majority of iLEXY embryos, but to a much lesser extent in embryos with the initial LEXY construct. At later developmental stages, iLEXY embryos displayed several other specific defects. When Twist-expressing cells were internalized, their subsequent dorsal migration was frequently impaired, and the mesodermal layer was often discontinuous with gaps along the anterior-posterior axis (Figure S4E). Some cells adapted a very elongated shape, with protrusions spanning the outer and inner cell layers. Toward the end of stage 10, Twist-expressing cells of the outer, but not the inner, cell sheet seemed to lose Twist expression prematurely (Figure S4E).

As muscle tissues are derived from the mesoderm, *twist*^*1*^ mutant embryos lack almost all muscles (Figure 3C). To examine this phenotype in iLEXY embryos, we stained embryos developed under blue light with an α-Tropomyosin antibody, a marker for all muscle types. At the end of embryogenesis, these blue-light-incubated embryos, but not dark-incubated or WT embryos, showed a dramatic reduction in muscles (Figure 3C). This loss of muscles was 100% penetrant and was most severe for *twist-mCherry-iLEXYs* and *twist-mCherry-iLEXYi* embryos, where only a few disorganized somatic muscles and hindgut muscles remained (Figure 3C). These muscle defects, as well as the germband retraction defects, closely resemble the *twist*^*1*^ loss-of-function mutant phenotype, although not fully. In contrast, the phenotypes observed with the initial LEXY construct were less severe and more variable (Figure 3C), indicating a less effective depletion of Twist from the nucleus. Placing such a “weaker allele” into a sensitized background could improve the severity of the phenotype. To test this, we crossed the initial *twist-LEXY* line with the *twist*^*1*^ allele to generate trans-heterozygous *twist-LEXY*/*twist*^*1*^ embryos (Figure 3C, bottom left panels). These embryos were viable when kept under safelight conditions, but their exposure to blue light led to a loss of muscles that is almost indistinguishable from *twist*^*1*^ homozygous mutant embryos (Figure 3C, bottom panels). Placing the optogenetic system into a sensitized background may, therefore, be a useful approach for proteins with lower nuclear depletion efficiency.

Taken together, iLEXY-mediated mislocalization of Twist causes developmental defects that phenocopy the genetic loss-of-function allele, demonstrating the effective perturbation of the TF’s function in living embryos. Since the highest nuclear depletion efficiency and most severe phenotypes were observed using iLEXY*s* and iLEXY*i*, we focused on these variants to further explore the roles of Twist during embryogenesis.

### Stage-specific depletion of Twist uncovers different requirements for Twist function

Given the early developmental defect in genetic *twist* mutant embryos, it has been very difficult to functionally dissect Twist’s role post-gastrulation. iLEXY now provides a unique opportunity to address such questions, by allowing embryos to gastrulate before removing Twist from the nucleus.

We first determined the developmental window where Twist is required. Twist is initially expressed in the presumptive mesoderm (stage 5) and then throughout the trunk and head mesoderm (stages 7–10), followed by a modulation of its expression into segmental high-and-low-expressing stripes (Figure S1A). Studies of *twist* loss-of-function mutants demonstrate that Twist is essential during gastrulation (which is initiated at stage 6 and completed during stage 7) but report conflicting time points for the subsequent requirement of Twist for embryonic survival. Using the same temperature-sensitive heteroallelic combination of *twist*^*ry50*^ and *twist*^*v50*^, Thisse et al. observed an effect of the restrictive temperature only between 2–4 h after egg lay (likely corresponds to stages 5–8) (Thisse et al., 1987). Baylies and Bate, on the other hand, found that Twist is also required during the subsequent period when Twist is expressed in stripes of high and low domains, with embryos having muscle defects that are incompatible with their survival into larval stages (Baylies and Bate, 1996). These discrepancies may be due to the extensive lethality of this temperature-sensitive allelic combination—only ∼30% of embryos are viable even at permissive temperatures (Thisse et al., 1987). Moreover, the *twist*^*ry50*^ and *twist*^*v50*^ alleles are only temperature-sensitive when placed in *trans* to each other, suggesting that only heterodimers of these two Twist mutant proteins, but not Twist^*ry50*^ and Twist^*v50*^ homodimers, are affected by the temperature shift (Castanon et al., 2001).

**Figure 4:**
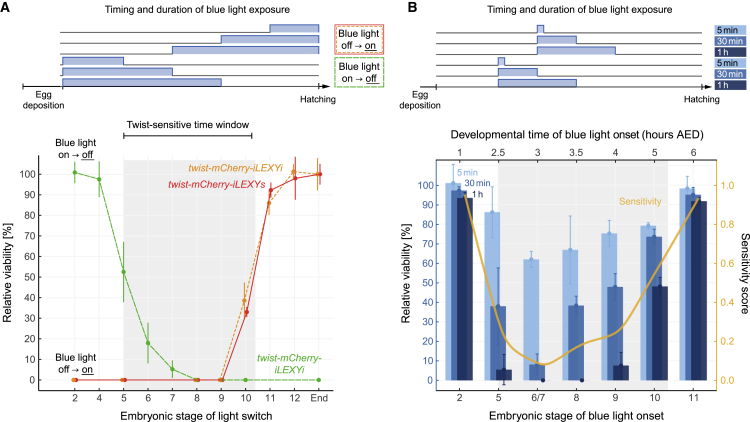
Mapping the precise developmental time window that requires Twist function (A) The temporal requirement of nuclear Twist for embryonic survival. Hatching assays (bottom panel) show the viability (mean ± SD) of homozygous *twist-mCherry-iLEXYi* and *twist-mCherry-iLEXYs* embryos. As illustrated (top panel), embryos were shifted from blue light “on” to dark (on → off = Twist nuclear “return”) or vice versa (blue light off → on = Twist nuclear depletion) at the developmental stage indicated. Rates are relative to the viability in the dark. Gray shading indicates the Twist-sensitive developmental time window. (B) Bar plot (bottom panel) showing relative viability rates (mean ± SD) of homozygous *twist-mCherry-iLEXYi* embryos depleted of nuclear Twist for different lengths of time. Embryos of indicated stages were exposed to 5 min (light blue bars), 30 min (blue bars), or 1 h (dark blue bars) of blue light (top panel). A sensitivity score (yellow line) calculated as the weighted mean (scaled from 0 to 1, 0 being most sensitive) summarizes the results. For each time point and condition shown in (A and B) at least two independent experiments were performed, with ~15–40 embryos each.

To precisely map the Twist-sensitive time window, we performed embryonic viability assays where Twist-iLEXY was first present and then depleted from the nucleus or was absent and then allowed to return to the nucleus (Figure 4A). For the depletion experiments (blue light off → on), we used both iLEXY*s* and iLEXY*i*, whereas for the “return” experiments (blue light on → off), we used iLEXY*i* taking advantage of its shorter recovery half-life (∼5 min, Figure 2B). In line with previous reports, we found that Twist is required for viability during the gastrulation stages 6 and 7, with the first effects of Twist depletion being noticeable at stage 5: embryos nuclear depleted of Twist from the beginning of embryogenesis until stage 5, 6, or 7 (blue light on → off) had a survival of ∼50%, 15%, and 5%, respectively (Figure 4A, green dashed line). In the opposite direction (blue light off → on), all embryos died when Twist was depleted from any stage between stages 1–9 until the end of embryogenesis; ∼35% and ∼90% of embryos survived when blue light was first applied from stage 10 and 11 onward, respectively (Figure 4A, solid red and dotted orange lines). Accordingly, Twist is required for embryonic survival during the post-gastrulation stages 8 and 9 and, to a lesser extent, during stage 10. Interestingly, Twist is not required at stage 11, despite the TF being still expressed, with particularly high expression in the ventral somatic muscle domain (Figure S1A).

Next, we assessed if embryos within this Twist-sensitive time window could recover from shorter depletion of the TF. We exposed *twist-mCherry-iLEXYi* embryos in which Twist has a recovery half-time of ∼5 min to blue light for periods of 5 min, 30 min, or 1 h, initiated at different developmental stages. Since some embryonic stages are shorter than 60 min (stages 5, 8 and 9) or even 10 min (stages 6 and 7), these blue light incubations often spanned multiple stages. The majority of embryos exposed to blue light for 1 h during stages 5–10 died, whereas younger and older embryos were mostly unaffected (Figure 4B). This maps to the identified Twist-sensitive time window (Figure 4A). Short depletions of Twist (5 min of blue light) were tolerated by more than half of the embryos throughout the Twist-sensitive time window. This indicates that Twist is able to resume its function after being reimported into the nucleus and that its absence is buffered, perhaps by the stability of already transcribed RNAs. Finally, blue light exposures for 30 min had a dramatic effect when initiated during the gastrulation stages 6 and 7 (and stopped during stage 8), but interestingly seemed less detrimental at earlier (stage 5) and later (stages 8–10) time points (Figure 4B). This suggests that genes dependent on Twist for their expression during the short period of gastrulation and/or immediately afterward (early-stage 8) are required for embryonic survival. These genes have likely not been transcribed earlier in embryogenesis and, therefore, have little or no residual transcripts that can buffer nuclear Twist depletion. A sensitivity score, calculated as the weighted mean of viability rates observed for the different conditions, revealed that the sensitivity to Twist depletion dramatically increases as embryos enter gastrulation but is highest during the transition from the gastrulation stages to stage 8. Once the embryos enter stage 8, the sensitivity slightly decreases but continues to stay high until stage 10, when a significant fraction of embryos (∼50%) survives even longer periods of Twist depletion (Figure 4B).

Taken together, the Twist-sensitive developmental time window mainly overlaps that of the TF’s pan-mesodermal expression, but not with its later expression in segmental stripes during stage 11. Embryos can partially recover from, or compensate for, shorter depletions of Twist (5 min of blue light) during any stage, but Twist function is least expendable during the transition from the gastrulation to the post-gastrulation stages.

### iLEXY can perturb a TF’s input at different temporal layers of a regulatory network

**Figure 5:**
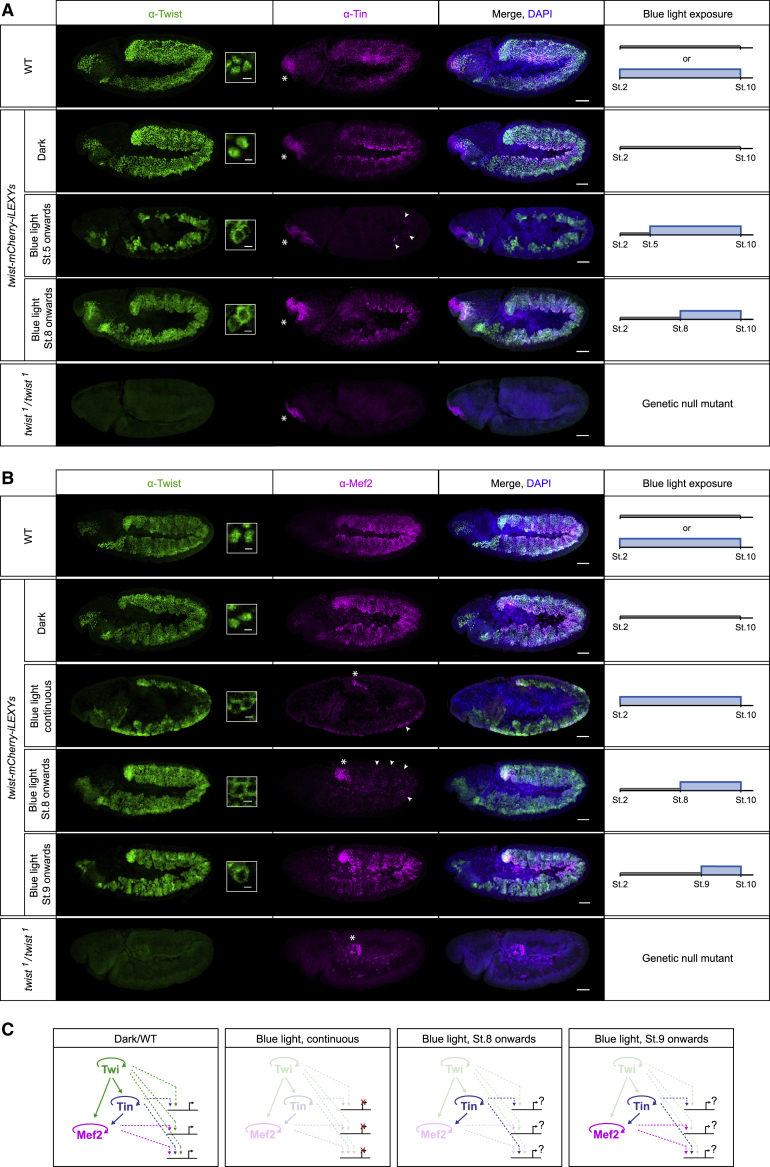
Temporal requirements of Twist for the expression of two direct target genes (A and B) Expression of Tinman (Tin) (A) and Mef2 (B) visualized by fluorescence immunostaining together with Twist and DAPI. Maximum intensity projections show stage 10 *twist-mCherry-iLEXYs* embryos incubated in the dark or blue light continuously or from the indicated developmental stage onward (right). Wild-type (WT) and *twist* loss-of-function mutant embryos (*twist*^1^/*twist*^1^) are shown for comparison. White asterisks indicate domains where Tin (A) or Mef2 (B) expression is independent of Twist. White arrowheads indicate cells expressing Tin or Mef2 sporadically. Dorsal up, anterior left; scale bars, 50 μm. Insets show Twist localization in individual cells. Scale bars, 5 μm. Note, embryos nuclear depleted of Twist continuously or from stage 5 onward, but not from stage 8 or 9 onward, show gastrulation defects. (C) Simplified mesodermal transcriptional network showing Twist, Tin, and Mef2 in WT or dark conditions and upon nuclear Twist depletion at the stages shown in (A) and (B).

Twist sits at the top of the regulatory network governing mesoderm development, where it regulates the expression of many genes, including TFs (Sandmann et al., 2007). Among these are Tinman (Tin) and Myocyte enhancer factor 2 (Mef2), required for the specification of tissues derived from the dorsal mesoderm and for muscle differentiation, respectively (Azpiazu and Frasch, 1993; Bodmer, 1993; Bour et al., 1995; Lilly et al., 1994). Once Tin and Mef2 are expressed, Twist co-occupies many regulatory elements with these TFs, forming potential feed-forward loops of enhancer regulation (Sandmann et al., 2007; Zinzen et al., 2009) (Figure 5C, left panel). However, the functional role of Twist in the regulation of these combinatorially bound enhancers has been difficult to discern, as in *twist* mutant embryos Tin and Mef2 are not expressed. iLEXY now enables the precise temporal perturbation of Twist after *tin* and *Mef2* expression have been induced, thereby disentangling the role of Twist in regulating these TFs’ expression versus the regulation of their common target genes.

To map the temporal requirement of Twist for Tin and Mef2 expression, we exposed *twist-mCherry-iLEXYs* embryos to blue light only after the embryos reached a certain developmental stage and performed immunostaining for the TFs. Consistent with the *twist*^*1*^ loss-of-function mutant phenotype, Tin and Mef2 were severely reduced upon continuous blue light exposure and only retained in domains where their expression is independent of Twist (Figures 5A and 5B) (Bodmer et al., 1990; Lilly et al., 1994; Taylor et al., 1995). In contrast, perturbation of Twist just after gastrulation (early-stage 8) allowed Tin to be expressed (Figure 5A) and perturbation from stage 9 onward facilitated Mef2 expression (Figure 5B). This illustrates that the individual steps in the feed-forward loops formed by Twist to Tin (Bodmer et al., 1990; Yin et al., 1997), and both Twist (Cripps et al., 1999; Nguyen and Xu, 1998; Taylor et al., 1995) and Tin (Cripps et al., 1999; Lovato et al., 2015) to *Mef2* can be discerned and confirms that Twist activates *tin* and *Mef2* in a temporally successive manner (Figure 5C).

Next, we obtained a more global view of Twist’s functional input at two time points of mesoderm development, before/during gastrulation (stages 5–8, 2–4 h after egg deposition [AED]) and after gastrulation (stages 9–10, 4–6 h AED), using RNA-seq. Although previous studies examined the transcriptional changes in *twist*^*1*^ loss-of-function mutant embryos at the latter time point (Furlong et al., 2001), secondary changes resulting from the misexpression of early Twist target genes and the failure of the presumptive mesoderm to invaginate will have contributed to the observed gene expression changes. Here, by perturbing Twist only after gastrulation, we can circumvent these secondary effects and separate Twist’s post-gastrulation function from its earlier role. Similarly, for the earlier time point (stages 5–8), where RNA-seq experiments on *twist* mutant embryos have not been previously performed, iLEXY provides unique advantages. In contrast to loss-of-function mutants, the iLEXY fusion lines are homozygous viable in the dark and, thereby, remove the need for sorting of homozygous embryos. This has four advantages: it (1) saves time, (2) increases the number of mutant embryos obtained from any single collection (rather than 25%, 100% of embryos have the genotype of interest), (3) facilitates the investigation of embryos at very early embryonic stages when fluorescent markers on the balancer chromosome are not yet visible (Casso et al., 2000), and (4) allows for a direct phenotypic comparison between genetically identical siblings, in the presence versus absence of blue light. Typically, phenotypes are compared between homozygous mutant and heterozygous embryos or another reference strain, which can lead to false positives due to background genetic differences between strains.

**Figure 6:**
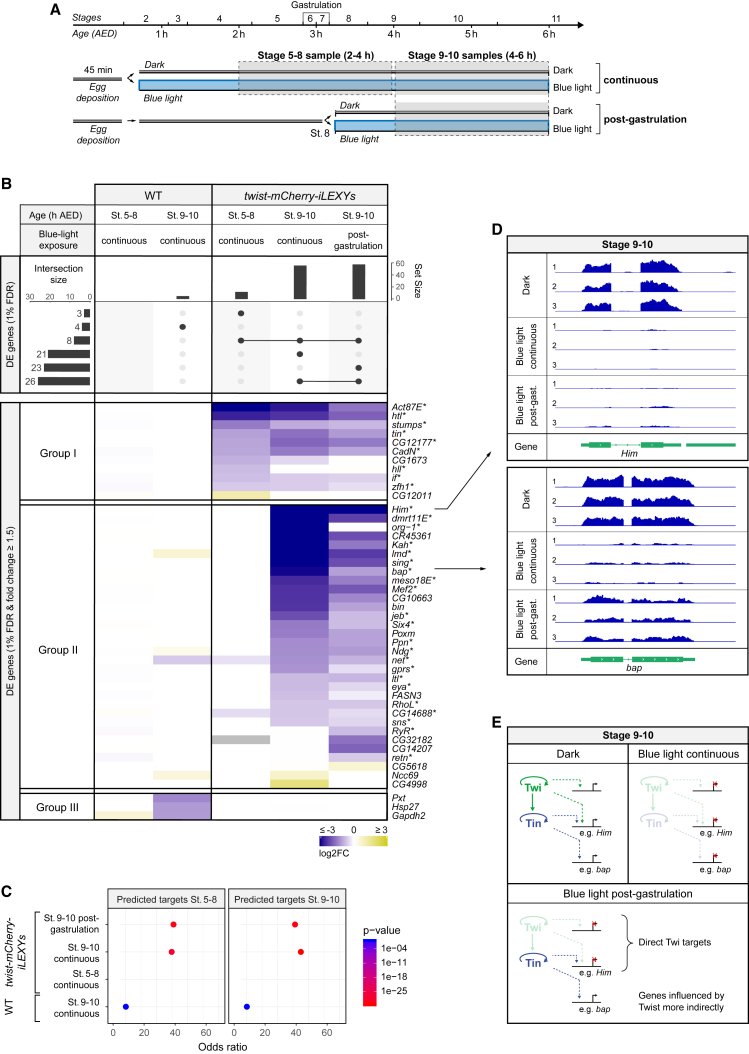
The molecular function of Twist before/during and after gastrulation (A) Embryo collections used for mRNA-seq. Homozygous *twist-mCherry-iLEXYs* embryos were collected and exposed to blue light immediately (“continuous”) or hand-sorted at early-stage 8 and exposed to blue light “post-gastrulation.” Embryos were aged to stages 5–8 (2–4 h AED) or stages 9–10 (4–6 h AED). mRNA-seq results were compared with sibling embryos kept in the dark. Wild-type (WT) embryos served as a control. (B) Differentially expressed (DE) genes (FDR < 0.01) upon continuous or post-gastrulation blue light exposure of WT and *twist-mCherry-iLEXYs* embryos at stages 5–8 and stages 9–10. Top row: UpSet plot showing total numbers of DE genes per sample (bar plot, top) and the intersection of DE genes of each sample sorted by the intersection size (bar plot, right). Bottom: Heat maps showing log_2_ fold change of the 43 DE genes with ≥ 1.5-fold change in *twist-mCherry-iLEXYs* embryos at stages 5–8 (Group I – early Twist-specific changes), at stages 9–10 but not stages 5–8 (Group II – late Twist-specific changes), and the 3 DE genes with ≥ 1.5-fold change in WT embryos at any time point (Group III –Twist-unspecific changes). Genes marked by an asterisk are predicted direct Twist targets based on ChIP and mutant expression data (Sandmann et al., 2007). (C) Significant enrichment of DE genes for predicted direct Twist target genes. Dot plots show odds ratios, colored by significance, of DE genes identified in the indicated time windows and high-confidence direct Twist target genes at stages 5–8 and stages 9–10 (Sandmann et al., 2007). No genes were DE upon blue light exposure of WT embryos at stages 5–8. (D) Expression of downstream genes is dependent on the timing of blue light exposure. Coverage tracks show mRNA-seq signal at the *Him* and *bap* loci of embryos in the dark and upon continuous or post-gastrulation nuclear Twist depletion at stages 9–10. All tracks are shown at the same scale. (E) Schematic illustrating the effect of conditional Twist perturbations on the expression of direct Twist target genes and genes influenced more indirectly. AED, after egg deposition. See also Figure S5 and Table S1.

We performed poly(A+)-RNA-seq from embryos at 2–4 h (stages 5–8, with stage 5 being the most frequent) and 4–6 h (mainly stages 9–10) of age that were depleted of nuclear Twist either continuously from 45 min after egg lay onward or immediately after gastrulation (early-stage 8 onward) and compared these with their dark-incubated siblings (Figure 6A). Three independent biological replicates were performed per condition. WT embryos served as a control for blue light illumination: genome-wide, zero or four genes were differentially expressed (DE) in these WT embryos at stages 5–8 and stages 9–10, respectively, indicating that blue light itself has little impact on gene expression (Figures 6B, Group III, S5G). In contrast, blue light exposure of *twist-mCherry-iLEXYs* embryos led to very specific gene expression changes. In total, 81 genes had significant (1% FDR) differential expression between dark or blue light in one or more condition, with 43 having ≥ 1.5-fold change (Figure 6B; Table S1). The majority of genes had reduced expression, reflecting the predominant role of Twist as a transcriptional activator (Figures S5A, S5C, and S5E). These DE genes are highly enriched for predicted Twist direct target genes (Sandmann et al., 2007) and genes in the vicinity of Twist binding (Zinzen et al., 2009) (Figures 6C and S5H), demonstrating the specificity of the observed response to the perturbation of the TF’s function.

The function of Twist before and during gastrulation is best reflected by DE genes at stages 5–8 (2–4 h) upon continuous nuclear Twist depletion (Figures 6B, Group I and S5B). Surprisingly, only 11 genes were DE during this time period (≥ 1.5-fold change at 1% FDR), 10 of which had reduced expression. All but one of these genes are also downregulated during gastrulation in loss-of-function mutants of the TF *snail* (stage 7) (Rembold et al., 2014). These genes are expressed early in the mesoderm (Bogaert et al., 1987; Karaiskos et al., 2017; Tomancak et al., 2002) and include TFs involved in mesoderm development (*tin* and *zfh1*), genes involved in mesoderm migration after gastrulation (*htl*, *stumps*, *if*), the long-chain-fatty-acid-CoA ligase encoding genes *heimdall* (*hll*), and the actin *Act87E* and *Cadherin-N* (*CadN*) genes. However, genes involved in gastrulation, namely *fog* (*folded gastrulation*), *T48* (*Transcript 48*), and *Traf4* (*NF-receptor-associated factor 4*) (Sweeton et al., 1991; Kölsch et al., 2007; Mathew et al., 2009, 2011; Seher et al., 2007), downstream of Twist were not detected as DE. As these genes have broader expression outside of the *twist*-expressing domain (Leptin, 1991; Costa et al., 1994; Mathew et al., 2011), their remaining Twist-independent expression could hinder DE detection in whole-embryo RNA-seq experiments. Indeed, *in situ* hybridization shows a dramatic downregulated in the expression of all three gastrulation genes (*fog*, *T48*, and *Traf4*) in the presumptive mesoderm upon nuclear Twist depletion, whereas their expression outside the Twist domain is unaffected, as also observed in *twist* loss-of-function mutant embryos (Figures S6A–S6D). This indicates that whole-embryo RNA-seq underestimates Twist responsive genes for genes expressed in regions outside the Twist domain.

The function of Twist after gastrulation is best reflected by DE genes at stages 9–10 (4–6 h) (Figures 6B, Group I and II, S5D, and S5F). These represent 78 DE genes (37 with ≥ 1.5-fold change), the majority of which were affected exclusively after gastrulation (70/78) and are involved in gene regulation and signaling. Many of these genes were identified with both continuous and post-gastrulation nuclear Twist depletion (Figure 6B), indicating that they are likely direct target genes. These include *Mef2* and *Hole in muscles* (*Him*), two genes known to be directly regulated by Twist (Cripps et al., 1999; Nguyen and Xu, 1998; Elwell et al., 2015) (Figures 6B (group II) and 6D, top panel). Other genes were mis-regulated only, or to a much larger extent, upon continuous nuclear Twist depletion and are more likely secondary effects. Examples include *bagpipe* (*bap*), *biniou* (*bin*), and *jelly belly* (*jeb*), three genes essential for visceral mesoderm development (Figure 6B). Our RNA-seq results, in combination with previous studies (Lee and Frasch, 2005), suggests that Twist influences *bap* expression, and thereby the Bap target gene *bin* (Zaffran et al., 2001), largely through the activation of *tin* at earlier stages (Figures 5A, 5C, 6E, and S6E). The temporal precision afforded by iLEXY can, therefore, help to distinguish between primary and secondary effects on target gene expression. The results also indicate that the key regulators of visceral mesoderm development (i.e., Tin, Bap, Bin, and Jeb) become largely independent of Twist after gastrulation (Figures 6B and S6E). This suggests that the post-gastrulation role of Twist is likely more important for somatic versus visceral muscle development. In line with this, embryos where Twist was depleted after gastrulation (from early-stage 8 onward) showed defects in all muscle tissues at the end of embryogenesis, but the somatic muscles were the most severely affected (Figure S7). A few embryos even developed a constricted midgut covered by visceral musculature similar to WT or dark-incubated embryos (Figure S7, top and bottom panel).

In summary, iLEXY enables the precise temporal depletion of an essential TF, Twist, which allowed us to determine its transcriptional response in different embryonic time windows. At early stages (stages 5–8), Twist acts to establish the mesoderm by facilitating gastrulation and the subsequent migration of mesodermal cells. In parallel, Twist activates TFs essential for mesoderm specification, regulating an extensive transcriptional network in a temporally successive manner. This second function begins before and during gastrulation with the expression of *tin* and *zhf1* but is most prevalent in the post-gastrulation stages.

## Discussion

While our ability to observe the dynamics of developmental processes has greatly advanced, tools to perturb the proteins underlying these processes at the required temporal resolution and efficiency are lacking in animal models. Here, we present iLEXY, which can fill this gap for nuclear proteins. While keeping the original system’s rapid activation kinetics, we greatly enhanced its nuclear depletion efficiency and light sensitivity, making iLEXY a powerful new tool to dissect nuclear protein function within their endogenous context in developing embryos. We demonstrate that iLEXY can be applied to a wide range of proteins *in vitro* and phenocopy a genetic loss-of-function mutant *in vivo*. Using Twist as a test case, we demonstrate the effectiveness of this system to temporally disentangle different roles of Twist during mesoderm development.

### A refined model of Twist function during mesoderm development

The investigation of Twist’s direct functional input during different stages of mesoderm development has been difficult using classic loss-of-function alleles: homozygous mutant embryos are difficult to distinguish from their heterozygous siblings at early embryonic stages due to the late expression of currently available GFP-expressing balancer chromosomes. During later stages, the absence of the mesoderm makes the examination of subsequent muscle development impossible. Both limitations are overcome by iLEXY, which we demonstrate can fill gaps in our knowledge about how this master regulator functions.

Nuclear depletion of Twist at early stages (stages 5–7) confirms that Twist is required during gastrulation (Figures 3B, S4D, 4A, 4B, and 7). However, contrary to expectations, the developmental time window where embryos are most sensitive to Twist depletion is the transition from the gastrulation stages 6/7 to the post-gastrulation-stage 8 rather than the transition from the pre-gastrulation-stage 5 to the gastrulation stages (Figure 4B). Approximately 15% of embryos depleted of nuclear Twist up until early gastrulation (stage 6, t_1/2_ ∼5 min) were still viable and hatched at the end of embryogenesis (Figure 4A). This could reflect the ability of embryos to partially compensate for early loss of Twist. At this stage, the expression of many Twist targets are also supported by the TF Snail, whereas other transcripts are maternally deposited (Zusman and Wieschaus, 1985; Costa et al., 1994; Urbansky et al., 2016). Only *twist* and *snail* double mutants display a complete loss of ventral furrow (Leptin and Grunewald, 1990), whereas overexpression of *snail* can partly rescue gastrulation in *twist* mutant embryos (Seher et al., 2007; Wong et al., 2014). Despite this potentially redundancy, most embryos depleted of nuclear Twist up until gastrulation die. In line with this, the expression of the gastrulation genes *fog*, *T48*, and *Traf4* is greatly reduced upon nuclear Twist depletion (Figures S6B–S6D), reflecting the importance of Twist for gastrulation.

Previous reports suggest that additional Twist target genes (potentially also non-essential genes) must exist that contribute to the gastrulation defect observed in *twist* loss-of-function mutants (Seher et al., 2007). None of the DE genes identified at stages 5–8 after Twist depletion have a reported role in gastrulation (Figure 6B). Nevertheless, it is tempting to speculate that the F-actin protein Act87E might. It is incorporated into cortical actin with a higher efficiency than any other *Drosophila* F-actins (Röper et al., 2005) and could contribute to morphogenetic processes within the mesoderm.

**Figure 7:**
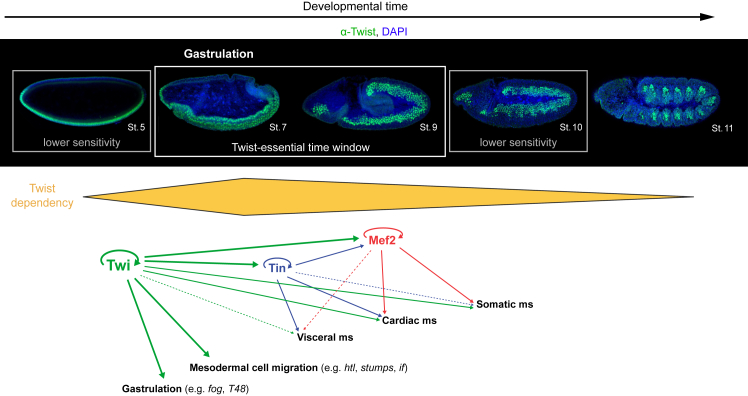
The role of Twist during embryonic mesoderm development The requirement of Twist at different embryonic stages. Nuclear depletion of Twist reveals three developmental time windows with different dependencies of Twist for embryonic viability (top panel, yellow; Figures 4A and 4B): a Twist-essential (stages 7–9, white box), a Twist-sensitive (stages 5 and 10, gray box), and a Twist-insensitive (stages 11 and 12) time window. Initially (stage 5–7), Twist is required for gastrulation and later for mesodermal cell migration by regulating the expression of corresponding genes. Twist also activates additional transcription factors in the downstream mesodermal gene regulatory network, including Tin and Mef2, in a temporally successive manner (Figure 5). Twist is no longer required for embryonic viability from stage 10 onward, when the role of Twist shifts from that of an essential mesodermal regulator to one likely contributing to the robustness of developmental programming.

After gastrulation, during stages 8 and 9, Twist is required for the activation of genes involved in mesodermal migration and subsequent development (Figure 7). This high sensitivity/essential time window is followed by a lower sensitivity window during which nuclear Twist depletion leads to reduced viability (stage 10) and, later on, by Twist-insensitive stages (stage 11–12) when the TF is no longer required for viability (Figures 4A and 7). The progressive decrease in sensitivity to nuclear Twist depletion from stage 8 onward reflects, at least in part, Twist target genes switching from an initial Twist-dependent to a more Twist-independent regulation of their expression (Figure 5). To our surprise, Twist starts to become dispensable for embryonic survival as early as stage 10, indicating that the somatic muscles develop sufficiently well for the embryos to hatch even when Twist is absent for the rest of embryogenesis. Since Twist is still expressed and binds to many enhancers at stage 11 (Sandmann et al., 2007; Zinzen et al., 2009), the TF likely still contributes (redundantly) to the expression of many genes but is no longer absolutely required. Our observed decrease in Twist-dependency over time thereby questions the proposed importance of high-Twist levels in the posterior part of each segment for somatic muscle development (Baylies and Bate, 1996), at least beyond stage 10.

Taken together, our results provide a refined view of Twist function. Although gastrulation is the TF’s first and most studied function, it is interestingly not the embryonic period most sensitive to nuclear Twist depletion. During this early developmental time window, Twist likely acts in parallel with other factors (e.g., Snail) that can partially compensate for its loss. In contrast, Twist is indispensable for the activation of the mesodermal transcriptional network during and directly after gastrulation. Once this network is established, the role of Twist slowly shifts from an essential master regulator to a downstream modulator that likely contributes to the robustness of gene expression. Going forward, iLEXY provides the tools to investigate the underlying molecular mechanism by which Twist is performing these diverse roles.

### iLEXY is a versatile tool to dynamically and effectively perturb the function of nuclear proteins in living embryos

The mislocalization strategy of LEXY, to transport nuclear proteins to the cytoplasm away from their location of action, has several advantages over other protein perturbation approaches: it is a one-component system, based on a small genetically encoded protein tag. Its induction by blue light is non-invasive and can be applied to a small number of cells or thousands of embryos. The perturbation has high-temporal dynamics and can be easily observed and readily quantified (Figure 2), and the approach can, in principle, be applied to any nuclear protein. However, a major hurdle that we were facing with the original LEXY construct was its low nuclear depletion efficiency and, thus, its low effectiveness. The phenotypes obtained when applied to Twist were variable and considerably less severe than the *twist* loss-of-function mutant (Figure 3C). To overcome this, we introduced slow-cycling *As*LOV2 variants (V416I and V416L), which increase both the system’s depletion efficiency (Figure 2I) and recovery time (Figures 2B and 2C). The resulting iLEXY*i* and iLEXY*s* variants can induce nuclear depletion of Twist to almost undetectable levels (Figure 2H), causing embryonic phenotypes similar to the characterized loss-of-function *twist* allele (Figure 3C). The increased recovery times of iLEXY*i* and iLEXY*s* also reduce the light-dose required to fully activate the system and thereby allow the light intensity (Figure 3A) and/or pulse-frequency to be reduced. This helps to decrease potential phototoxicity and, in the latter case, also facilitates sample handling and longer image acquisitions of live embryos in between blue light laser pulses. Although iLEXY*i* and iLEXY*s* are very similar in their activation kinetics and effectiveness, they differ in their recovery half-times, making iLEXY*s* (t_1/2_ ∼ 50 min) best suited for constant depletions and iLEXY*i* (t_1/2_ ∼ 5 min) for recovery experiments and repeated depletions.

iLEXY is a very versatile tool, compatible with both live imaging of single embryos and bulk experiments with thousands of embryos, including genomic and biochemical assays. In contrast to genetic mutants or protein-degradation systems, cells expressing the iLEXY fusion protein can be easily identified after the protein’s depletion, by visualizing the now cytoplasmic protein either directly using the mCherry fluorophore (Figures 2D–2F) or using immunostaining (Figures 2H and S2E). This facilitates, for example, cell tracing, cell sorting, and the observation of cell and tissue morphologies that might result from the induced perturbation (Figures 3B, S4D, and S4E). For most of the experiments presented here, we fully activated iLEXY depletion. However, the system could, in principle, also be controlled quantitatively to deplete nuclear proteins to different levels by varying the intensity or duration of blue light illumination. For Twist, we observed a gradual decrease in embryonic viability as the blue light intensity increased, with the slope being smallest for the *twist-LEXY* allele (Figure 3A).

Given that the initial LEXY construct was developed in mammalian cells, iLEXY can likely be applied to a wide range of model systems. Therefore, we envisage that the improved optogenetic tool will shed new light on the function and mechanism of many pleiotropic regulators and dynamic developmental processes.

### Limitations of the study

Although iLEXY can, in principle, be applied to any nuclear protein, proteins that are firmly anchored in the nucleus (for example H2B; Niopek et al., 2016), might be a limitation. To quickly determine if the system works for a given protein of interest, we developed an iLEXY transient transfection-based mislocalization assay in *Drosophila* cell culture. Applying this system to 12 nuclear proteins, indicates that the system can be applied to many, but not all proteins (Figure S3). Therefore, we strongly recommend testing iLEXY*i* or iLEXY*s* with the nuclear protein of interest in cell culture prior to the more time-consuming task of generating knockin animals with the endogenously tagged locus. When transferring the desired fusion protein into animals, it is also worth testing iLEXY variants with and without mCherry (or with another easily detectable tag such as HA) as we found that mCherry can sometimes interfere with protein function. This can result in lines that, in the dark, are homozygous lethal with the mCherry-iLEXY*s* tag, while being homozygous viable using iLEXY*s* without mCherry.

In the *Drosophila* embryo and at the stages investigated here (stages 5–11), blue light penetration is sufficient to fully activate iLEXY (Figure S2F), likely also due to the enhanced light sensitivity of the slow-cycling variants. Because each nuclear protein’s affinity and retention in the nucleus is different, the strength and duration of blue light required for maximal depletion should be assessed. In addition to quantifying the depletion by immunostains, ChIP-seq can also be used where possible to assess potential residual chromatin binding. This could provide interesting insights about the nuclear protein's binding properties to different genomic regions. Lastly, we note that all systems tested (the initial LEXY construct as well as the iLEXY variants) display some dark-state activity, meaning that even in the absence of blue light, a small proportion of molecules exist in the Jα helix unfolded state and is, therefore, exported from the nucleus (Figures 2I and S2F). This dark-state activity is highest for LEXY and iLEXY*i* and lowest for iLEXY*s*. Although, in our experience, these small changes in protein levels are usually tolerated and do not cause any obvious phenotypes, a respective dark control should always be conducted.

## STAR★Methods

### Key resources table

**Table:** 

REAGENT or RESOURCE	SOURCE	IDENTIFIER
**Antibodies**		

Alexa Fluor conjugated secondary antibodies	Thermo Fisher Scientific	Various
Chicken anti-β-Galactosidase IgG	Abcam	Cat# ab9361
Guinea pig anti-Twist	Michael Levine, Princeton University	N/A
Mouse anti-Elav IgM	DSHB	Cat# ELAV 9F8A9
Mouse anti-Lamin	DSHB	Cat# ADL101-s
Rabbit anti-DsRed	TaKaRa	Cat# 632496
Rabbit anti-Mef2 IgG	Eileen Furlong laboratory, EMBL	N/A
Rabbit anti-Tin IgG	Manfred Frasch laboratory, University of Erlangen-Nuremberg	N/A
Rat anti-Tropomyosin IgG1	Babraham	Cat# P6694
Sheep-anti-Digoxigenin-POD	Roche	Cat# 1207733
Wheat Germ Agglutinin, Alexa Fluor 680	Thermo Fisher Scientific	W32465

**Bacterial and virus strains**		

XL1-Blue Competent cells	Custom made. Eileen Furlong laboratory, EMBL	N/A

**Chemicals, peptides, and recombinant proteins**		

DNase I recombinant, RNase-free	Roche	Cat# 04716728001
DRAQ5 fluorescent probe	Thermo Fisher	Cat# 62251
Halocarbon oil 10S	Voltalef, VWR	Cat# 24627.188
Halocarbon oil 27	Sigma	Cat# H8773-100ML
Paraformaldehyde 16% solution (Methanol-free	Agar Scientific	Cat# 287577
Prolong Gold antifade reagent with DAPI	Thermo Fisher	Cat# P36931
T4 DNA ligase	NEB	Cat# M0202L
T4 Polynucleotide Kinase PNK	NEB	Cat# M0201S
TRI Reagent	Sigma-Aldrich	Cat# T9424-100ML

**Critical commercial assays**		

Agencourt RNAClean XP kit	Beckman Coulter	Cat# A63987
AMPureXP beads	Beckman Coulter	Cat# A63881
CloneAmp HiFi PCR Premix	Clontech	Cat# 639298
DIG RNA Labelling Mixture	Roche	Cat# 11277073910
FuGENE HD Transfection Reagent	Promega	Cat# E2311
NEBNext Poly(A) mRNA Magnetic Isolation Module	NEB	Cat# E7490S
NEBNext Ultra Directional RNA Library Prep Kit for Illumina	NEB	Cat# E7420S
Quickchange II Site-Directed Mutagenesis kit	Stratagene	Cat# 200523
TSA Plus Cy3 and Fluor kit	Perkin Elmer	Cat# NEL753001KT

**Experimental models: Cell lines**		

*D. melanogaster*: Cell line Kc167	DGRC	RRID:CVCL_Z834
*D. melanogaster*: Cell line Schneider 2 (S2)	DGRC	RRID:CVCL_TZ72

**Deposited data**		

RNA-seq sequence data	This study	E-MTAB-9034

**Experimental models: Organisms/strains**		

*D. melanogaster*: yw y[1]w[1118]	Bloomington Drosophila Stock Center	RRID: BDSC_6598
*D. melanogaster*: double-blancer If/CyO; MKRS, Sb[1] /TM6B, Tb[1]	Eileen Furlong laboratory, EMBL	N/A
*D. melanogaster*: *twist*^1^ cn[1] twi[1] bw[1] speck[1]/CyO P{ftz-lacZ}	Castanon et al., 2001	N/A
*D. melanogaster*: piggyBac w[1118]; Herm{3xP3-ECFP,alphatub-piggyBacK10}M6	Bloomington Drosophila Stock Center	RRID: BDSC_32071
*D. melanogaster*: *vasa-Cas9* w[1118]; PBac{y[+mDint2]=vas-Cas9}VK0002	Bloomington Drosophila Stock Center	RRID: BDSC_51324
*D. melanogaster*: VK33 y[1] M{RFP[3xP3.PB] GFP[E.3xP3]=vas-int.Dm}ZH-2A w^∗^; PBac{y^+^-attP-3B}VK00033	Bloomington Drosophila Stock Center	RRID: BDSC_24871
*D. melanogaster*: H2B-iRFP670 y[1]w[1118]; +; {tub-H2BiRFP670( w^+^)}VK00033(y^+^)	This study	N/A
*D. melanogaster*: *twist*-*LEXY*: w[1118]; TI{RFP[DsRed.3xP3.Scarless]=TI}twi[LEXY]	This study	N/A
*D. melanogaster*: *twist*-*LEXY*, w/o DsRed w[1118]; twi[LEXY]	This study	N/A
*D. melanogaster*: *twist*-*mCherry-LEXY* w[1118]; TI{RFP[DsRed.3xP3.Scarless]=TI}twi[mCherry-LEXY]	This study	N/A
*D. melanogaster*: *twist*-*mCherry-iLEXY(N414L)* w[1118]; TI{RFP[DsRed.3xP3.Scarless]=TI}twi[mCherry-LEXY[N414L]]	This study	N/A
*D. melanogaster*: *twist*-*mCherry-iLEXYi* TI{RFP[DsRed.3xP3.Scarless]=TI}twi[mCherry-LEXY[V416I]]	This study	N/A
*D.melanogaster*: *twist*-*mCherry-iLEXYs* TI{RFP[DsRed.3xP3.Scarless]=TI}twi[mCherry-LEXY[V416L]]	This study	N/A

**Oligonucleotides**		

twist-gRNA1_for: CTTCGCAGCACCAGAAGGCATAG	This study	N/A
twist-gRNA1_rev: AAACCTATGCCTTCTGGTGCTGC	This study	N/A
twist-gRNA2_for: CTTCGTTAGACTATGACATTAAAT	This study	N/A
twist-gRNA2_rev: AAACATTTAATGTCATAGTCTAAC	This study	N/A
LEXY(N414L)_for: CTGTTTGTCATTACTGACCCAAGATTG	This study	N/A
LEXY(N414L)_rev: CTTCTCAATACGTTCAAGTGTAGTAG	This study	N/A
LEXY(V416I)_for: ATCATTACTGACCCAAGATTGCC	This study	N/A
LEXY(V416L)_for: CTGATTACTGACCCAAGATTGCCAG	This study	N/A
LEXY(V416)_rev: AAAGTTCTTCTCAATACGTTCAAGTG	This study	N/A

**Recombinant DNA**		

Plasmid: pAc5.1/V5-His (Version C)	Invitrogen	Cat# V411020
Plasmid: pAc-twist-mCherry-iLEXY*s*	This study	N/A
Plasmid: pAc-Mef2-mCherry-iLEXY*s*	This study	N/A
Plasmid: pAc-tin-mCherry-iLEXY*s*	This study	N/A
Plasmid: pAc-bin-mCherry-iLEXY*s*	This study	N/A
Plasmid: pAc-bap-mCherry-iLEXY*s*	This study	N/A
Plasmid: pAc-prg-mCherry-iLEXY*s*	This study	N/A
Plasmid: pAc-cdk9-mCherry-iLEXY*s*	This study	N/A
Plasmid: pAc-NelfE-mCherry-iLEXY*s*	This study	N/A
Plasmid: pAc-TfIIB-mCherry-iLEXY*s*	This study	N/A
Plasmid: pAc-RpII140-mCherry-iLEXY*s*	This study	N/A
Plasmid: pAc-fs(1)h-mCherry-iLEXY*s*	This study	N/A
Plasmid: pAc-Sin3A-mCherry-iLEXY*s*	This study	N/A
Plasmid: pDN122 - NLS-mCherry-AsLOV2-NES21	Addgene	ID 72655
Plasmid: pU6-BbsI-chiRNA	Addgene	ID 45946
Plasmid: pU6-twist-gRNA1	This study	N/A
Plasmid: pU6-twist-gRNA2	This study	N/A
Plasmid: pHD-pScarless-DsRed	Addgene	ID 64703
Plasmid: pHD-LH-twist-mCherry-LEXY(N414L)-RH	This study	N/A
Plasmid: pHD-LH-twist-mCherry-iLEXY*i*-RH	This study	N/A
Plasmid: pHD-LH-twist-mCherry-iLEXY*s*-RH	This study	N/A
Plasmid: pOTB7-twist, BDGP EST clone AT15089	DGRC	Cat# 11695
Plasmid: pFlc-1-tin, BDGP EST clone RE01329	DGRC	Cat# 7896
Plasmid: pOT2-htl, BDGP EST clone LD32130	DGRC	Cat# 5217
Plasmid: pOT2-T48, BDGP EST clone GM18993	DGRC	Cat# 12509
Plasmid: pBS SK(-)-Traf4, BDGP EST clone LD20987	DGRC	Cat# 3650
RNA *in situ* probe: *fog*	Maria Leptin laboratory	N/A

**Software and algorithms**		

apeglm (version 1.4.1)	Zhu et al., 2019	https://bioconductor.org/packages/apeglm
DESeq2 (version 1.22.1)	Love et al., 2014	https://github.com/mikelove/DESeq2
Fiji	Schindelin et al.,2012	https://fiji.sc
flyCrispr target finder	Gratz et al., 2014	http://targetfinder.flycrispr.neuro.brown.edu
gplot2 (version 3.0.0)	Warnes et al., 2020	CRAN.R-project.org/package=gplots
R	R Core Team, 2019	https://r-project.org

**Other**		

Illumina NextSeq 500 High Output Flow Cell	Leica	N/A
Leica SP8	Leica	N/A
HC Pl APO CS2 20x/0.75 IMM objective	Leica	N/A
HC PL Apo CS2 100x/1.4 oil objective	Leica	N/A
Zeiss LSM780	Zeiss	N/A
Plan Apochromat 20x/0.8 objective	Zeiss	N/A
C-Apochromat 63x/1.4oil DIC	Zeiss	N/A
Blue light LED (WL-SMDC SMD Mono-color Ceramic LED Waterclear)	Würth	Cat# 150353BS74500
Arduino Uno ATmega328P microcontroller	Arduino	Barcode: C8058333490090
Lee Filter 135 Deep Golden Amber	Huss Licht & Ton	Cat# LF050135
Fibreboard boxes	National Lab	Cat# KA1100BU1
Lumox foil, oxygen-permeable membrane	Sarstedt	Cat# 94.6077.317

### Resource availability

#### Lead contact

Further information and requests for resources/reagents should be directed to Eileen Furlong (furlong@embl.de) who will coordinate their provision.

#### Materials availability

The fly lines used in this study were generated by the authors as described in the methods section and are maintained for the community by the Furlong lab at EMBL. Only costs to cover post and packaging will be requested. Non-commercial antibodies used in this study were published previously.

### Experimental model and subject details

#### *D. melanogaster* genetics and husbandry

All fly lines were raised on standard food between 18°C and 25°C. Embryo collections were performed on apple juice agar plates with yeast paste at 25°C and 60% humidity. *Drosophila* embryos homozygous for the LEXY- or iLEXY-tagged *twist* alleles are blue-light-sensitive and had to be protected from exposure to any blue light, including blue light containing daylight and white light. Maintenance, handling, and embryo collections with these fly lines were, therefore, carried out under safelight conditions. To this end, all light sources, including lamps of microscopes used for embryo staging, were covered with amber light filter foil (Guglielmi and De Renzis, 2017). For the transport of light-sensitive embryos or fly lines, fibreboard boxes were used.

Kockins into the endogenous *twist* locus were carried out using the scarless CRISPR-Cas9 gene editing technology (https://flycrispr.org/scarless-gene-editing/). CRISPR donor and gRNA plasmids were mixed at a 2:1:1 molar ratio (Donor:gRNA1:gRNA2) with a total DNA concentration of ∼500 ng/μl. This mixture was injected into *vasa-Cas9* embryos by the EMBL *Drosophila* injection service. Positive transformants were identified by DsRed expression in the eyes of adult F1 flies and used to establish stable stocks. The GFP-marked *vasa-Cas9* allele was removed by crosses to yw or a double-balanced fly line. The precise integration of the respective tag and the integrity of the *twist* locus were confirmed by PCR amplification and subsequent sequencing of the CRISPR allele, from outside of the left homology arm to outside of the right homology arm (Gratz et al., 2015). The expression and cellular localization of tagged Twist were validated by immunostaining against Twist and, where applicable, mCherry (Figure 1C). The DsRed marker cassette of LEXY and iLEXY fly lines was removed by a single cross to piggyBac transposase expressing flies, resulting in a scarlessly edited genome.

For live imaging experiments, LEXY and iLEXY fly lines were crossed to flies carrying an iRFP670 (Shcherbakova and Verkhusha, 2013) tagged *dm*H2B marker gene, which was integrated by PhiC31 integrase-mediated transgenesis into the VK33 landing site. The yw fly line, used for the outcross of injected flies, served as a wild-type (WT) reference. Homozygous *twist* loss-of-function (null) embryos were obtained from a *twist*^*1*^/*CyO ftz-lacZ* fly line (Castanon et al., 2001) and identified by the absence of the lacZ gene product expressed from the balancer chromosome by anti-β-Galactosidase staining. The *twist*^*1*^ allele (initially called *twist*^*ID96*^) (Nüsslein-Volhard et al., 1984; Simpson, 1983) produces *twist* mRNA, but no Twist protein.

Important note: Most experiments presented in this paper were carried out with LEXY and iLEXY fly lines still carrying the DsRed marker cassette. In the case of the *twist* locus, the presence of this marker cassette did not influence embryonic viability or light sensitivity (Figure S4A). Nevertheless, we strongly recommend the removal of the marker when integrating mCherry along with the LEXY or iLEXY cassette. mCherry and DsRed are highly similar in sequence (∼90%) and can, at a very low frequency, cause the loss of the LEXY sequence in between them by homologous recombination.

#### *D. melanogaster* cell culture model

*Drosophila* Kc167 or S2 cells were cultured according to standard protocols (Baum and Cherbas, 2008; Yang and Reth, 2012). Transient transfection of cells with different mCherry-iLEXY*s* constructs was performed either by electroporation according to (Cherbas et al., 1994) using 10 μg of plasmid DNA or using the FuGENE HD Transfection Reagent with slight modifications from previous protocols (Yang and Reth, 2012). In short, high-quality plasmid DNA was isolated using the QIAGEN Plasmid Plus Midi Kit. Transfections were carried out in 6-well plates in a cell culture volume of 2 ml. For each transfection 1 μg plasmid DNA and 4 μl FuGENE HD Transfection Reagent were pre-mixed and incubated in 100 μl serum- and antibiotic-free medium before being added to the cells. The choice of the transfection method did not influence the outcome of the mislocalization assay and was always monitored using Twist as a positive control.

### Method details

#### Generation of LEXY and iLEXY constructs

The LEXY and mCherry-LEXY cassettes were PCR-amplified from the NLS-mCherry-LEXY (pDN122) plasmid (Niopek et al., 2016) and expanded N-terminally by a 10 amino-acid serine-glycine linker (L: DSAGSAGSAG). For the expression of iLEXY-fusion proteins in *Drosophila* tissue culture cells, the L-mCherry-LEXY fragment was integrated into the XhoI/BstBI site of the expression vector pAc5.1/V5-His. The coding sequence of different nuclear proteins of interest, amplified from EST clones or *Drosophila* cDNA, was inserted into the XhoI site in-frame and upstream of the L-mCherry-LEXY cassette. For the integration of LEXY or iLEXY into the endogenous *twist* locus *in vivo*, CRISPR donor and CRISPR gRNA vectors were constructed as follows: gRNAs targeting the *twist* stop codon (gRNA1) and a TTAA site 174 bp downstream of the *twist* coding sequence (gRNA2) were designed using the flyCRISPR Target Finder (tools.flycrispr.molbio.wisc.edu/targetFinder/) (Gratz et al., 2014). gRNA sequences were generated by annealed oligo cloning and inserted into the BbsI site of the pU6-BbsI-gRNA vector (Gratz et al., 2013) according to (Gratz et al., 2015). For the homology repair template, the *twist* right homology (RH) arm (spanning the TTAA and its 1173 bp downstream sequence) was amplified from the targeted fly line and integrated into the SapI site of the pHD-DsRed-Scarless donor vector. A fusion product of the *twist* left homology (LH) arm (2000 bp region directly upstream of gRNA1 cleavage site, amplified from the targeted fly line), the L-LEXY or L-mCherry-LEXY fragment, and the genomic region between the gRNA1 cleavage site and the TTAA sequence were generated by overlap extension PCR and inserted into the AarI site of the same plasmid (Gratz et al., 2015).

Improved LEXY (iLEXY) variants were generated by the integration of single point mutations into the *As*LOV2 domain (Figure 1B). Further modifications, in the NES or the linker region between the mCherry and the LEXY sequence, were added to the iLEXY*s* variant (Figure 1B). In each case, PCR-mediated site-directed mutagenesis was performed on the cell culture expression or the CRISPR donor vector, following one of two different strategies. (1) Either, the entire template plasmid was PCR-amplified using the CloneAmp HiFi PCR Premix according to the manufacturer's instructions with non-overlapping oligonucleotides carrying the desired mutation or integration at the 5′ end (see key resources table). The resulting PCR products were phosphorylated and ligated using the T4 Polynucleotide Kinase PNK and the T4 DNA ligase according to the manufacturer's instructions. (2) Alternatively, the Quickchange II Site-Directed Mutagenesis kit was used according to the manufacturer’s instructions. All generated LEXY and iLEXY fusion constructs were sequence verified.

#### Live imaging and laser blue light induction

For live imaging experiments, collected *Drosophila* embryos were dechorionated for 2 min in 8% NaOCl (50% household bleach, diluted in water). Using a fine paintbrush, dechorionated embryos were transferred onto a piece of apple-juice agar plate and aligned and oriented under a binocular stereo-microscope with transmitted light. The hydrophobic side of an oxygen-permeable membrane was pre-coated with embryo glue prepared from heptane dissolved double-sided adhesive tape (Garcia and Gregor, 2018). By carefully pressing its coated side against the apple-juice agar, the embryos were then transferred to the membrane. The membrane was mounted into a membrane-holding slide (Garcia and Gregor, 2018), 3D-printed by the EMBL mechanical workshop. Finally, embryos were covered with halocarbon oil 27 and a coverslip of 22 x 22 mm (No. 1.5). For light-sensitive embryos, the described procedure was performed under safelight conditions.

Embryos expressing Twist-mCherry-LEXY or -iLEXY and the nuclear marker H2B-iRFP670 were live imaged on a Zeiss LSM 780 confocal laser-scanning microscope using a Plan-Apochromat 20x/0.8 or C-Apochromat 63x/1.40 oil DIC objective. mCherry and iFRP were imaged sequentially and excited with 561 nm or 633 nm laser light, respectively. For time-course experiments, mCherry and iFRP images were acquired every 30 s with image acquisition taking approximately 7 s per channel. In between intervals of image acquisition, cells within the region of interest were either left in the dark or scanned 10 times with a 458 nm laser (scan speed of 6) using the ‘bleaching’ setting of the ZEN software.

Note: Live imaging of *Drosophila* embryos was complicated by the movement of cells along the Z-axis, the overall weak signal, the rapid bleaching of the mCherry fluorophore, and the alternation between image acquisition and blue light illumination. Therefore, a compromise between image quality, imaging speed, signal brightness, and signal bleaching is generally made. As a consequence, we have likely underestimated the fold-change in Twist nuclear depletion observed by live imaging. These issues are overcome in fixed embryos, likely contributing to the differences in nuclear/cytoplasmic ratio changes observed between experiments performed on live and fixed embryos (Figures 2A–2C and 2I). Similarly, Niopek et al., 2016 obtained a two-fold difference in nuclear depletions between different microscopy techniques.

#### Blue light induction using a custom LED box

Bulk blue light illumination of *Drosophila* embryos was achieved using a custom-made, programmable LED based blue light illumination box built by the EMBL electronic workshop with help from the EMBL mechanical workshop. In this box, two boards with 28 LEDs each were placed above and below a transparent stage to allow sample illumination from two directions (Figure 2G). A microcontroller was used to set the LED intensity, pulse frequency, and pulse-width-modulation (pwm). Unless otherwise stated, 2 s blue light pulses with an intensity of 70% were intermitted by 1 s breaks.

Embryos were collected for 45 min at 25°C and, where appropriate, aged until the desired stage. Embryos were dechorionated for 2 min in 8% NaOCl (50% household bleach, diluted in water) and rinsed with water, before being subjected to one of two different incubation strategies. For incubations up to 6 h after egg deposition, embryos were simply spread in a transparent Petri dish containing PBTr (PBS + 0.05 % Triton-X100) and then exposed to blue light or kept in the dark. The amount of PBTr was sufficient to submerge the embryos but prevented extensive floating of embryos above and below each other (∼2.5 ml PBTr in a 10 mm dish). Embryo clumps and overcrowding was avoided to ensure sufficient oxygen supply and even illumination. Alternatively, for longer incubations, embryos were spread on a piece of apple-juice agar plate and transferred to a coverslip coated with embryo glue (Garcia and Gregor, 2018) by carefully pressing the coverslip with the coated side against the agar. Embryos were subsequently covered with halocarbon oil 10S and incubated in the LED box or in the dark. Space of at least one embryo length was left between embryos to ensure sufficient gas exchange. If required, embryos of specific stages were selected in either case.

To fix embryos within the LED box, i.e. during blue light illumination, we adapted the fixation procedures described by Trcek et al., 2017 and Rothwell and Sullivan, 2007 with slight modifications. Formaldehyde-saturated heptane was prepared at least one day before use as follows: Equal volumes of heptane and fixative (4% methanol-free paraformaldehyde in PBS) were combined in a glass bottle and mixed at least three times by vigorous shaking. The bottle was then stored at room temperature, protected from light. The upper heptane phase was used for subsequent fixations. Embryos incubated in a Petri dish with PBTr (see above) were poured into a collection basket, drained on tissue paper, and transferred into a glass vial containing 1.5 ml freshly prepared fixative (see above) and 1.5 ml formaldehyde-saturated heptane. Embryos were shaken vigorously for 15 s and, without further shaking, incubated in the LED box (or in the dark) for 20 min. Afterwards, embryos were devitellinized and stored in methanol at -20°C.

To fix embryos after longer bulk blue light incubations, embryos first had to be recovered from the halocarbon oil. This procedure was adapted from previous protocols describing the fixation of microinjected *Drosophila* embryos (Allan, 2000; Juarez et al., 2013; Minden et al., 2000). In short, the oil was drained off the coverslip by carefully leaning it against a rack and blotting the end with tissue paper. To dissolve the glue and release the embryos, heptane was rinsed over the coverslip using a glass pipette. Embryos were collected in a glass Petri dish containing heptane. Embryos were washed once with fresh heptane, transferred to a glass vial, and fixed in a fixative/heptane mixture (1 ml fixative, see above, and 2 ml heptane) for 20 min on a shaker at maximum speed. After devitellinization, embryos were stored in methanol at -20°C.

Importantly, all steps, except the incubations within the LED box, were performed under safelight conditions. Dark-incubated and WT embryos were subjected to the same experimental procedures to control for any effects that these might have on the molecular and development processes investigated. Dark incubated embryos were further used to control for potential effects of the optogenetic system’s dark state activity (see also Discussion and Figure 2I).

Please note that in this study, embryos were always dechorionated before their exposure to blue light for the following reasons: Firstly, the removal of the chorion improves the transparency of embryos and therefore very likely the penetration of blue light. Secondly, dechorionation allows for a better comparison to corresponding live imaging experiments. And thirdly, dechorionation improves the visibility of embryo morphologies and thereby facilitates precise staging. However, our tests indicate that, at least in the case of iLEXY*s*, similar muscle loss phenotypes are obtained for *twist-mCherry-iLEXY* embryos exposed to blue light without prior removal of the chorion. The omission of dechorionation simplifies the above-described experimental procedures as it eliminates the need to cover embryos with PBTr or halocarbon oil.

#### Embryo viability assays

Embryo viability was examined by scoring the hatching rate of embryos into first instar wandering larvae. Following three 1 h pre-lays, embryos were collected for 45 min at 25°C and, where appropriate, aged until the desired stage. Embryos were then washed with water and dechorionated for 2 min in 8% NaOCl (50% household bleach, diluted in water). Afterwards, embryos were rinsed with water to remove residual bleach, drained on tissue paper, and transferred to a piece of apple-juice agar. Under a binocular stereo-microscope, approximately 50 embryos were aligned using a dissecting needle. Specific stages were hand-selected based on embryo morphology according to Campos-Ortega and Hartenstein, 1997. The appearance of furrows and folds, easily visible under a stereoscope, was used for the selection. Importantly, space of at least one embryo length was left between individual embryos to ensure sufficient gas exchange. Afterwards, embryos were transferred to a coverslip coated with embryo glue, prepared from heptane dissolved double-sided adhesive tape (Garcia and Gregor, 2018), and covered with halocarbon oil S10. The coverslip was placed on a glass slide and incubated in the LED box under blue light illumination or kept in the dark at 25°C.

For the rapid gastrulation stages 6 and 7 (which last only 10 min each), embryos were prepared on a coverslip and covered by halocarbon oil in advance. For stage 6, embryos were exposed to blue light immediately after the cephalic furrow became visible. For stage 7, the additional appearance of transverse folds was awaited. Embryos of older or younger stages were removed in either case.

After 24 h, the embryos were transferred into a humidified chamber and hatching first instar larvae were collected with a dissecting needle approximately 30 h and 48 h after egg deposition. The number of hatched, unhatched, and unfertilized eggs was determined and used to calculate the viability rate of embryos. Importantly, all steps were performed under safelight conditions, unless stated otherwise, and embryos were only exposed to blue light at the indicated times.

#### Immunostaining and *in situ* hybridization

Whole-mount *Drosophila* embryo immunostaining and fluorescent *in situ* hybridization (FISH) were carried out according to standard protocols (Patel, 1994) and as described previously (Schor et al., 2018). As primary antibodies, chicken anti-β-Galactosidase (1:500) was used to identify embryos carrying *Cyo ftz-lacZ* balancer chromosomes, rabbit anti-DsRed (1:200) to stain mCherry, mouse anti-Elav (1:300) and rat anti-Tropomyosin (1:4000) to stain the nervous system and musculature, respectively, of stage 15-16 embryos, and guinea pig anti-Twist (1:300), rabbit anti-Mef2 (1:400, Furlong lab), and rabbit anti-Tinman (1:1000) were used to stain for the respective transcription factors. Alexa Fluor conjugates were used as secondary antibodies (1:500). The Wheat Germ Agglutinin (WGA) Alexa Fluor 680 conjugate (1mg/ml 1:100) or mouse anti-lamin (1:300) was used to stain nuclear membranes. Digoxigenin-labeled RNA *in situ* probes for *tin*, *twist*, *htl*, *T48,* and *Traf4* were prepared from corresponding EST clones using the DIG RNA Labelling Mixture. The digoxigenin-labeled *fog* probe was obtained from Maria Leptin’s laboratory. mRNA expression was visualized using these *in situ* probes together with anti-Digoxigenin-Peroxidase (1:2000) and TSA Plus Fluorescence kits. Stained Embryos were mounted in ProLong Gold Antifade Mountant with DAPI and imaged on a Leica SP8 confocal laser-scanning microscope with an HC Pl APO CS2 20x/0.75 IMM or an HC PL Apo CS2 100x/1.4 oil objective.

#### Cell culture based mislocalization assays

Protein mislocalization assays in *Drosophila* tissue culture were performed on cells transiently transfected with the target plasmid (see above). Approximately 48 h after transfection and 30 min before imaging, 4 μl DRAQ5 fluorescent probe was diluted in 500 μl medium and added to each well to stain the nuclei. Cells were subsequently kept in the dark. The mCherry and DRAQ5 signals of transiently transfected cells were imaged on a Zeiss LSM 780 confocal laser-scanning microscope using a C-Apochromat 63x/1.40 oil DIC objective. Images were taken before and after irradiating cells within a region of interest repeatedly with a 458 nm laser using the ‘bleaching’ setting of the ZEN software (10 iterations each with a scan speed of 6).

#### mRNA-seq

For mRNA-seq experiments, wild-type (WT) and homozygous *twist-mCherry-iLEXYs* flies were grown in medium-sized cages. Following three 1 h pre-lays, embryos were collected for 45 min before being subjected to one of two different blue light exposure schemes (Figure 6A). For continuous blue light exposure, embryos were immediately dechorionated using 50% bleach and spread in transparent Petri dishes containing PBTr (PBS + 0.05 % TritonX-100). Older embryos that resulted from retention of fertilized eggs by females were removed by rapid hand-sorting under a stereo-microscope. Half of the embryos were then kept in the dark, while the other half was exposed to blue light in the LED illumination box. For light exposure post-gastrulation, collected embryos were aged for 2.5 h in the dark before being dechorionated. On a piece of apple-juice agar plate and under a stereo-microscope, early stage 8 embryos were hand-selected before being spread in Petri dishes containing PBTr and transferred to the LED illumination box or kept in the dark. Finally, for all samples, embryos of consecutive collections were combined into a 2-4 h or 4-6 h AED (after egg deposition) collection, blotted dry, transferred into a reaction tube, snap-frozen in liquid nitrogen, and stored at -80°C. A small portion of embryos from each sample was formaldehyde fixed and devitellinized to allow subsequent staging. Samples at 2-4 h AED contained mainly stage 5-8 embryos (with stage 5 being the most frequent) and samples at 4-6 h AED mainly contained stage 9-10 embryos. All steps were performed at 25°C and, apart from the incubation in the LED box, under safelight conditions. Two or three independent collections were performed for each condition for WT and *twist-mCherry-iLEXYs* embryos, respectively.

For RNA extraction, embryos were homogenized in TRI Reagent using pestles and a cordless motor and total RNA was extracted according to the manufacturer's instructions. DNA was digested using RNase-free DNase I, followed by an RNA clean-up step using the Agencourt RNAClean XP kit according to the manufacturer's protocol. Poly(A+) mRNA isolation and fragmentation were performed using the NEBNext Poly(A) mRNA Magnetic Isolation Module according to the manufacturer's instructions (manual E7420, Chapter 1). 1 μg total RNA was used as starting material and the protocol for 200 bp RNA inserts was followed. RNA-seq libraries were prepared using the NEBNext Ultra Directional RNA Library Prep Kit for Illumina according to the manufacturer's instructions (manual E7420, Chapter 1). For library enrichment, 12 PCR cycles were used. Indexed RNA-seq libraries were multiplexed in an equimolar ratio and paired-end sequenced with a read length of 75 bp on an Illumina NextSeq 500 High Output Flow Cell. For the WT fly line, two biological replicates, and for the *twist-mCherry-iLEXYs* line, three biological replicates were performed per condition. The resulting RNA-seq sequence data have been submitted to the EMBL-EBI European Nucleotide Archive (E-MTAB-9034).

### Quantification and statistical analysis

#### Quantification of nuclear/cytoplasmic Twist

Measurements of the Twist or Twist-mCherry signal in the cytoplasm and the nucleus of individual cells were performed in Fiji. For live imaging experiments, the H2B-iFRP670 signal was used to determine the position of nuclei and the mCherry signal was used to quantify Twist. To determine the nuclear and the cytoplasmic fraction of mCherry, two regions of interest (ROIs) were manually drawn in both the nucleus and cytoplasm of each cell, at each time point investigated. The area and the raw integrated density (RawIntDen) of these regions were measured. For each time point, the same measurements were also taken from regions not overlapping any mCherry-expressing cells (background (BG)) and from regions overlapping the entire group of cells investigated (AllCells) to determine the background fluorescence and the bleaching effect, respectively. The relative nuclear and cytoplasmic mCherry fluorescence was then calculated as follows: First, the mean RawIntDen was calculated for the nucleus and cytoplasm of each cell and each time point and divided by the area measured. The resulting average RawIntDen/μm^2^ was then background subtracted and multiplied with the bleaching factor calculated for the respective time point (equations (1) and (2)). Finally, the nuclear and the cytoplasmic fluorescence obtained for each time point were divided by the nuclear fluorescence of the first time point (t_0_) (equations (3)). Similarly, relative nuclear/cytoplasmic fluorescence ratios were obtained. Cells undergoing mitosis during the imaging time course were excluded from the analysis.

(Equation 1) $$\text{Bleaching factor }t_{x}=\left(\frac{{\text{RawIntDen}}_{AllCells}}{{\text{Area}}_{AllCells}}–\frac{{\text{ RawIntDen}}_{BG}}{{\text{Area}}_{BG}}\right)t_{x}/\left(\frac{{\text{RawIntDen}}_{AllCells}}{{\text{Area}}_{AllCells}}–\frac{{\text{ RawIntDen}}_{BG}}{{\text{Area}}_{BG}}\right)t_{0}$$

(Equation 2) $$\text{Fluorescence }t_{x}=\;\left(\frac{\text{Mean RawIntDen}}{\text{Area}}\;–\frac{{\text{ RawIntDen}}_{BG}\;}{{\text{Area}}_{BG}}\right)t_{x}\times \text{ Bleaching factor }t_{x}\;$$

$$\text{Relative fluorescence nucleus }t_{x}=\text{ Fluorescence nucleus }t_{x}/\text{ Fluorescence nucleus }t_{0}$$

(Equation 3) $$\text{Relative fluorescence cytoplasm }t_{x}=\;\text{Fluorescence cytoplasm }t_{x}\;/\;\text{Fluorescence nucleus }t_{0}$$

The nuclear and the cytoplasmic fraction of Twist in cells of fixed samples were quantified from anti-Twist immunostaining. Wheat Germ Agglutinin (WGA) or anti-lamin staining were used to determine the position of nuclei. The area and the RawIntDen of manually drawn ROIs in the cytoplasm and the nucleus of each cell were determined. For each cell, measurements from three consecutive Z-planes were then used to calculate the average nuclear and cytoplasmic RawIntDen per μm^2^. Measurements from non-Twist expressing cells were used to determine the background fluorescence. Background subtracted average RawIntDen per μm^2^ were used to calculate the nuclear/cytoplasmic fluorescence ratios per cell. Cells undergoing mitosis, identified using DAPI staining, were excluded from the analysis.

#### Differential gene expression analysis

Differential gene expression analysis from RNA-seq data was performed using DESeq2 (Love et al., 2014). Calculated log_2_ fold-changes were shrunk using the approximate posterior estimation for generalized linear models approach (apeglm) (Zhu et al., 2019). Differential genes were identified using an FDR cutoff of < 0.01 and an absolute shrunken log_2_ fold-change of ≥ 0.01. To determine the enrichment of previously described Twist target genes in the gene set with differential expression, odds ratios were generated. Here, two sets of previously described Twist target genes were used: (1) Putative direct Twist target genes, based on having both a ChIP peak (using ChIP-chip data) and differential expression in *twist* mutants (using microarray data), were extracted from Sandmann *et al.* 2007. Genes that were determined to be high confidence targets at 2-4 h and/or 4-6 h were merged and old gene IDs converted to the current *Drosophila* annotation using Flybase. Only genes with a one-to-one translation were used (Figure 6C). (2) Twist ChIP peaks (from high density tiling arrays) were taken from Zinzen *et al.,* 2009. The coordinates of ChIP peaks were converted from dm2 to dm6 using the liftOver tool and chan files from UCSC and genes with the nearest transcriptional start site to the peak were used for enrichment analysis (Figure S5H). Statistical significance was calculated using a one-sided Fisher exact test. To obtain Gene Ontology (GO) term enrichment, the R library GOstat version 2.52.0 was used with the *Drosophila* genome annotation from R library org.Dm.eg.db version 3.5.0. We used biological process function trees and performed a hypergeometric test. The p values were adjusted for multiple testing using Benjamini-Yekutieli correction.

## Data Availability

The RNA-seq data generated in this study has been dposited to the EMBL-EBI European Nucleotide Archive and are publicly available as of the date of publication - ENA: E-MTAB-9034. No custom code other than scripts to connect the publicly available programs described in the STAR Methods section were used in this study.

## Supplemental information

Document S1. Figures S1–S7

Table S1. Differential gene expression analysis results, related to Figure 6

Document S2. Article plus supplemental information
