# Supplementary material for: Extremely rapid and reversible optogenetic perturbation of nuclear proteins in living embryos
Source: Dev Cell. 2021 Aug 23;56(16):2348–2363.e8. doi: 10.1016/j.devcel.2021.07.011 (PMC8387026; doi:10.1016/j.devcel.2021.07.011)
Supplement: Document S1. Figures S1–S7 [file mmc1.pdf]

Developmental Cell, Volume *56*

Supplemental information

Extremely rapid and reversible optogenetic perturbation of nuclear proteins in living embryos

Anna C. Kögler, Yacine Kherdjemil, Katharina Bender, Adam Rabinowitz, Raquel Marco-Ferreres, and Eileen E.M. Furlong

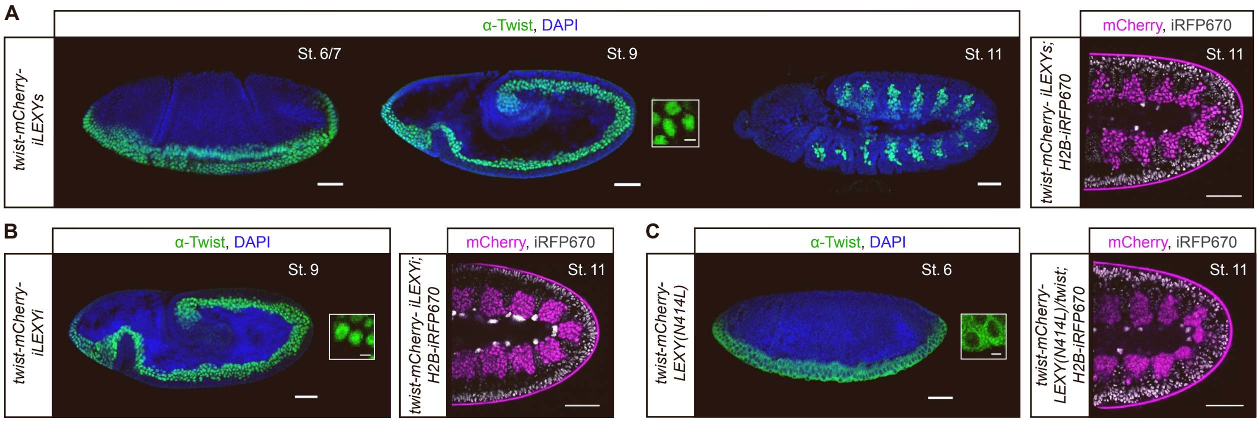

Figure S1 (related to Figure 1): Twist expression and subcellular localization in embryos upon fusion with different iLEXY variants

Expression and subcellular localization of Twist-mCherry-iLEXY*s* **(A)**, Twist-mCherry-iLEXY*i* **(B)**, and Twist-mCherry-LEXY(N414L) **(C)** in dark-incubated embryos at the indicated stage visualized by immunostaining against Twist (green, left panel) and by live imaging of mCherry (magenta, right panel). Nuclei are stained with DAPI (blue, left panel) or marked by the expression of Histone H2B-fused iRFP670 (grey, right panel). Dorsal is up and anterior to the left. Scale bars, 50 µm. Insets show individual cells. Scale bar, 5 µm. Please note that the embryo shown in C, right panel, is heterozygous. All fly lines, except the ones expressing Twist-mCherry-LEXY(N414L), are homozygous viable under safelight conditions (Figure S2G).

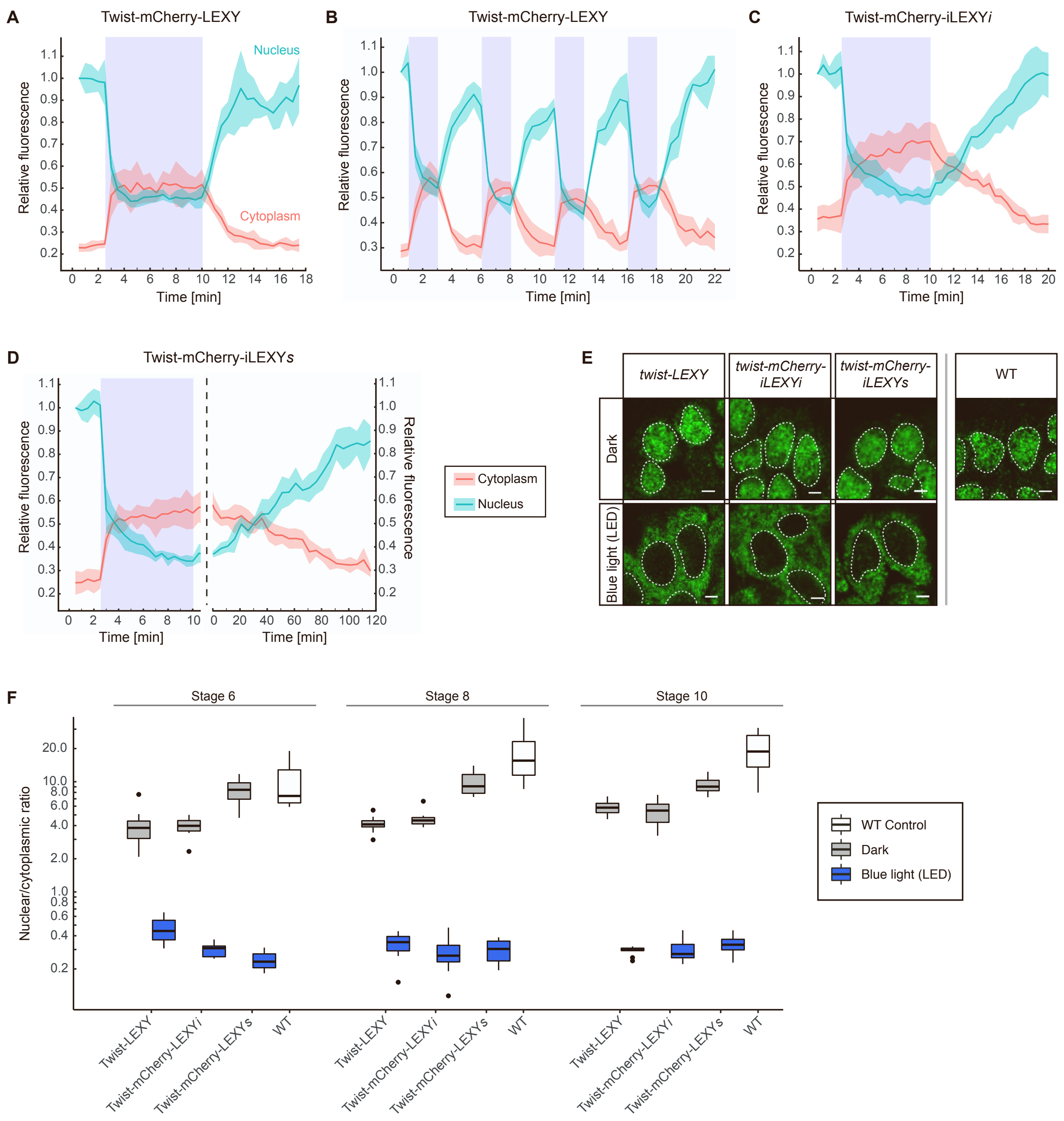

G

Fly line	Dark				Daylight	Blue light (LED)			Kinetics	
	Viability of		Main protein localization	Twist-specific expression pattern	Viability of homozygous line	Viability of		Main protein localization	Half-times ($t_{1/2}$)	
	homozygous line	heterozygous line				homozygous line	heterozygous line		activation	recovery
WT	Yes	Yes	nuclear	Yes	Yes	Yes	Yes	nuclear	/	/
twist-LEXY	Yes	Yes	nuclear	Yes	Yes	No	Yes	cytoplasmic	?	?
twist-mCherry-LEXY	Yes	Yes	nuclear	Yes	Yes	No	Yes	cytoplasmic	< 30 sec	~1.5 min
twist-mCherry-LEXY(N414L)	No	Yes	cytoplasmic	Yes	No	No	Yes	cytoplasmic	/	/
twist-mCherry-iLEXYi	Yes	Yes	nuclear	Yes	No	No	Yes	cytoplasmic	~30 sec	~5 min
twist-mCherry-iLEXYs	Yes	Yes	nuclear	Yes	No	No	Yes	cytoplasmic	~30 sec	~50 min

Figure S2 (related to Figure 2): Blue-light-induced nuclear depletion of Twist with LEXY and different iLEXY variants

(A-D) Blue-light-induced changes in the localization of Twist-mCherry-LEXY (**A, B**), Twist-mCherry-iLEXY*i* (**C**), and Twist-mCherry-iLEXY*s* (**D**) observed by live confocal imaging of mCherry in stages 9-10 embryos. Groups of mCherry expressing cells were imaged over a time course of blue light laser irradiation (indicated by the blue rectangles) and subsequent recovery in the dark. Mean (line) ± sd (shading) of nuclear (green) and cytoplasmic (red) mCherry fluorescence are shown relative to the nuclear fluorescence at the first time point. For Twist-mCherry-iLEXY*s* (D), activation and recovery were recorded separately. See also Movies S1 and S2. Note, the same time-lapse images were used to calculate the nuclear/cytoplasmic mCherry ratio (shown in Figures 2A-C) and here to calculate the mCherry levels in the nucleus and cytoplasm. **(E)** High-resolution images of α-Twist immunostaining in stage 10 wild-type embryos (WT; right) or in dark and LED blue light-incubated stage 10 embryos homozygous for *twist* tagged with the indicated LEXY variant. Blue light incubation was performed for 60 min in the LED blue light box. Images were used to quantify the light-induced nuclear depletion of Twist achieved using this setup (Figures 2I and S2F). Nuclei are outlined by dashed lines. Scale bars, 2 µm. **(F)** Twist localization quantified as nuclear/cytoplasmic fluorescence ratios of α-Twist signal from immunostained embryos. Measurements were performed at stages 6, 8, and 10 in wild-type (WT) embryos (white boxplot) or *twist-LEXY*, *twist-mCherry-iLEXYi,* and *twi-mCherry-iLEXYs* embryos incubated in the dark or for 60 min under LED blue light (grey and blue boxplots, respectively). The reduced nuclear/cytoplasmic ratio in dark-incubated LEXY-tagged embryos compared to WT embryos likely results from LEXY's dark state activity (see also Discussion). Differences in the nuclear/cytoplasmic ratio between developmental stages, which are also observed for the WT, likely reflect normal changes in Twist expression levels over this developmental time. **(G)** Table summarizing the expression, localization, and kinetics of Twist fused to the indicated LEXY variants and the viability of respective fly lines under different light conditions. Activation and recovery kinetics could only be measured for constructs carrying mCherry and are therefore undetermined in *twist-LEXY* embryos.

A

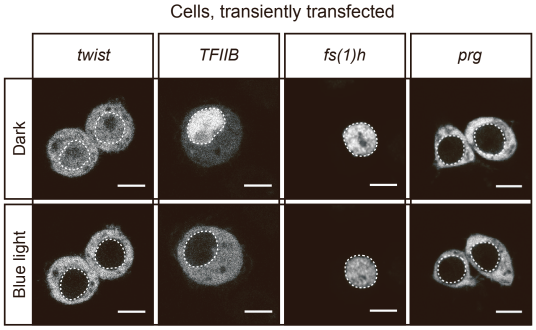

B

Nuclear protein	Mislocalization to the cytoplasm upon blue light exposure	Protein function	Predicted MW [kDa]
Twist (Twi)	Yes	TF	54
Mef2	Yes	TF	53 - 64
Tinman (Tin)	Yes	TF	46
Biniou (Bin)	Yes	TF	71
Bagpipe (Bap)	Yes	TF	42
Piragua (Prg)	No (cytoplasmic)	TF	64

Nuclear protein	Mislocalization to the cytoplasm upon blue light exposure	Protein function	Predicted MW [kDa]
Cdk9	No (cytoplasmic)	Cyclin dependent kinase	47
NelfE	No	Negative elongation factor	32
TfIIB	Yes	General transcription factor	34
RpII140	No	RNA polymerase II subunit	134
Fs(1)h	No	Transcriptional repressor	110 - 206
Sin3A	Yes	Chromatin regulator	186 - 221

Figure S3 (related to Figure 2): iLEXY-mediated nuclear depletion of different nuclear proteins

(A) Live confocal imaging of mCherry in transiently transfected *Drosophila* tissue culture cells expressing mCherry-iLEXY*s* tagged to different nuclear proteins. Images were taken before (top panel) and during (bottom panel) the irradiation of cells with a blue light laser. Dashed lines mark the position of nuclei. Scale bar, 5 µm. Please note that some constructs (e.g. Twist) show both nuclear and cytoplasmic localization in the dark, likely representing a cell culture artifact caused by overexpression. Nevertheless, the blue light response of the fusion protein can be accessed. Other constructs (e.g. Prg-mCherry.iLEXY*s*) already localize to the cytoplasm in the dark, indicating that the NES might be constitutively accessible. **(B)** Table summarizing the proteins tested for nuclear export using transient transfection in *Drosophila* cell lines. The molecular function and predicted molecular weight (MW) (FlyBase, calculated using the BioPerl SeqStats module) of the proteins are indicated.

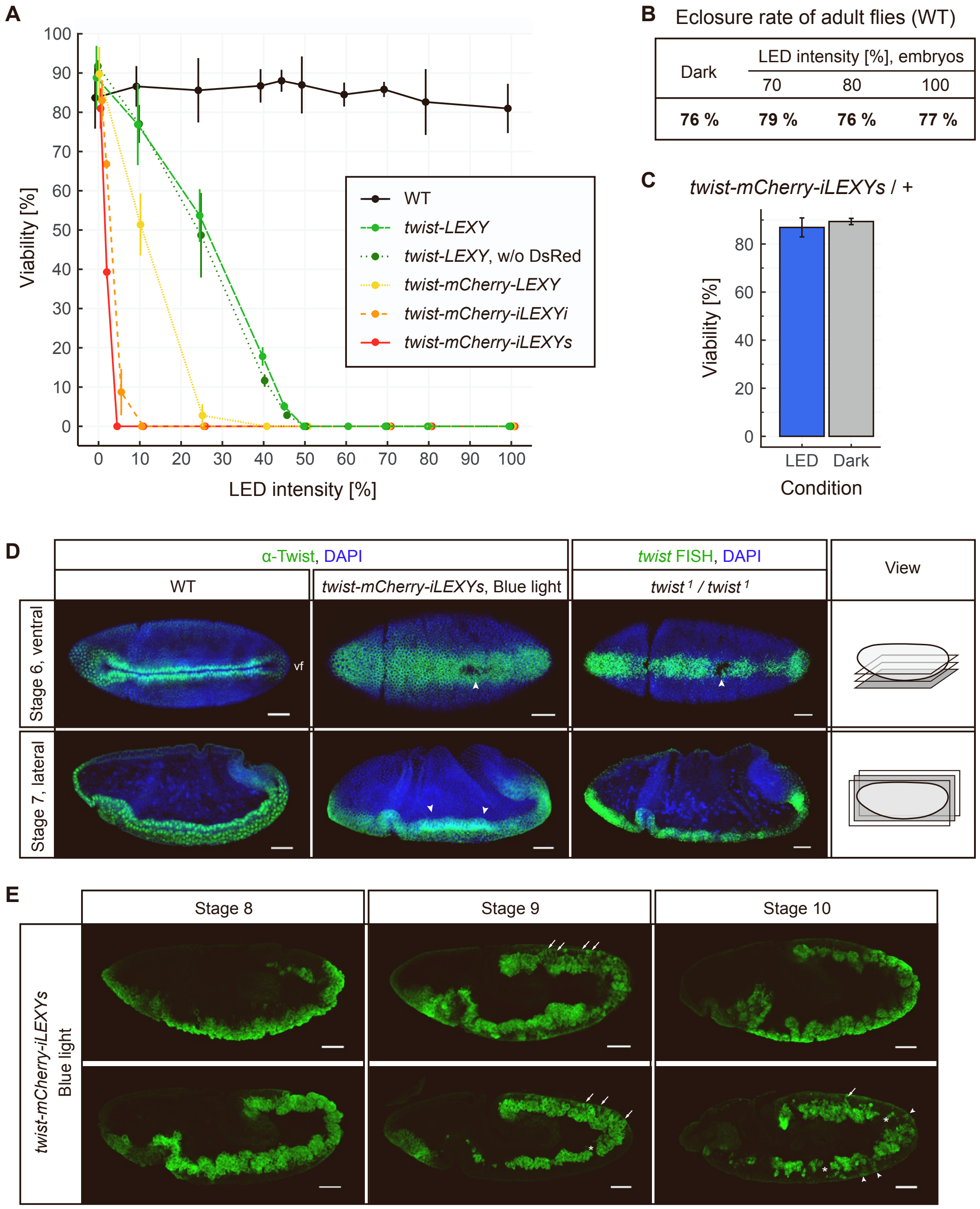
A
Viability [%]
LED intensity [%]
WT
twist-LEXY
twist-LEXY, w/o DsRed
twist-mCherry-LEXY
twist-mCherry-iLEXYi
twist-mCherry-iLEXYs
B
Eclosure rate of adult flies (WT)
Dark
LED intensity [%], embryos
70
80
100
76 %
79 %
76 %
77 %
C
twist-mCherry-iLEXYs / +
Viability [%]
LED
Dark
Condition
D
α-Twist, DAPI
twist FISH, DAPI
View
WT
twist-mCherry-iLEXYs, Blue light
twist 1 / twist 1
Stage 6, ventral
Stage 7, lateral
vf
E
Stage 8
Stage 9
Stage 10
twist-mCherry-iLEXYs
Blue light

Figure S4 (related to Figure 3): iLEXY-mediated nuclear Twist depletion is embryonic lethal and causes diverse mesodermal defects

(A) Viability of wild-type (WT) embryos and embryos homozygous for *twist* alleles tagged with the indicated LEXY variant at increasing LED blue light intensities. Embryos were exposed to the indicated LED intensity in the LED blue light box continuously throughout development. The graph shows the mean ± sd of at least two independent assays quantifying the percentage of embryos that hatched into wandering first instar larvae. The DsRed marker cassette, integrated during the CRISPR/Cas genome editing, was removed in the indicated fly lines (w/o DsRed). **(B)** Survival of larvae, hatched from dark- and blue-light-incubated WT embryos, to adulthood. WT larvae that hatched from the viability assay presented in (A) were followed until the eclosure of adult flies. **(C)** Viability of embryos heterozygous for the *twist-mCherry-iLEXYs* allele in the presence and absence of blue light (LED and Dark, respectively). Mean ± sd of four hatching assays performed from two different crosses are shown. Similar hatching rates were observed for each condition, indicating that cytoplasmic Twist does not influence the embryo's survival. **(D)** Gastrulation of cells of the ventral furrow (vf) in WT, homozygous blue-light-incubated *twist-mCherry-iLEXYs*, and homozygous *twist*^{1}/*twist*^{1} loss-of-function (null) mutant embryos. α-Twist immunostaining or *twist* FISH (green) are shown as maximum intensity projections of ventral view stage 6 embryos and lateral view stage 7 embryos. Locally restricted but deep invaginations (arrowheads) can be observed in a subset of nuclear Twist depleted and mutant embryos (for mutant embryos see also Seher *et al.*, 2007). Please note that *twist*^{1}/*twist*^{1} mutant embryos produce *twist* mRNA, but no Twist protein, allowing the staining of the corresponding *twist* domain by FISH, but not by immunofluorescence. Nuclei are stained with DAPI (blue). Anterior is left in each panel and dorsal is up in the bottom panel. Scale bar, 50 µm. **(E)** Phenotypes of homozygous stages 8-10 *twist-mCherry-iLEXYs* embryos exposed to blue light continuously throughout development. Maximum intensity projections show α-Twist immunostaining. Due to the iLEXY-mediated nuclear depletion, Twist is located primarily in the cytoplasm and thereby facilitates the visualization of cell membrane protrusions, as seen spanning the external and internal cell sheets (arrows). Asterisks mark regions in the trunk mesoderm of stage 9 and 10 embryos that completely miss Twist-expressing cells. Arrowheads indicate cells in the external cell sheet of a late stage 10 embryo expressing low levels of Twist. Dorsal is up and anterior is left. Scale bars, 50 µm.

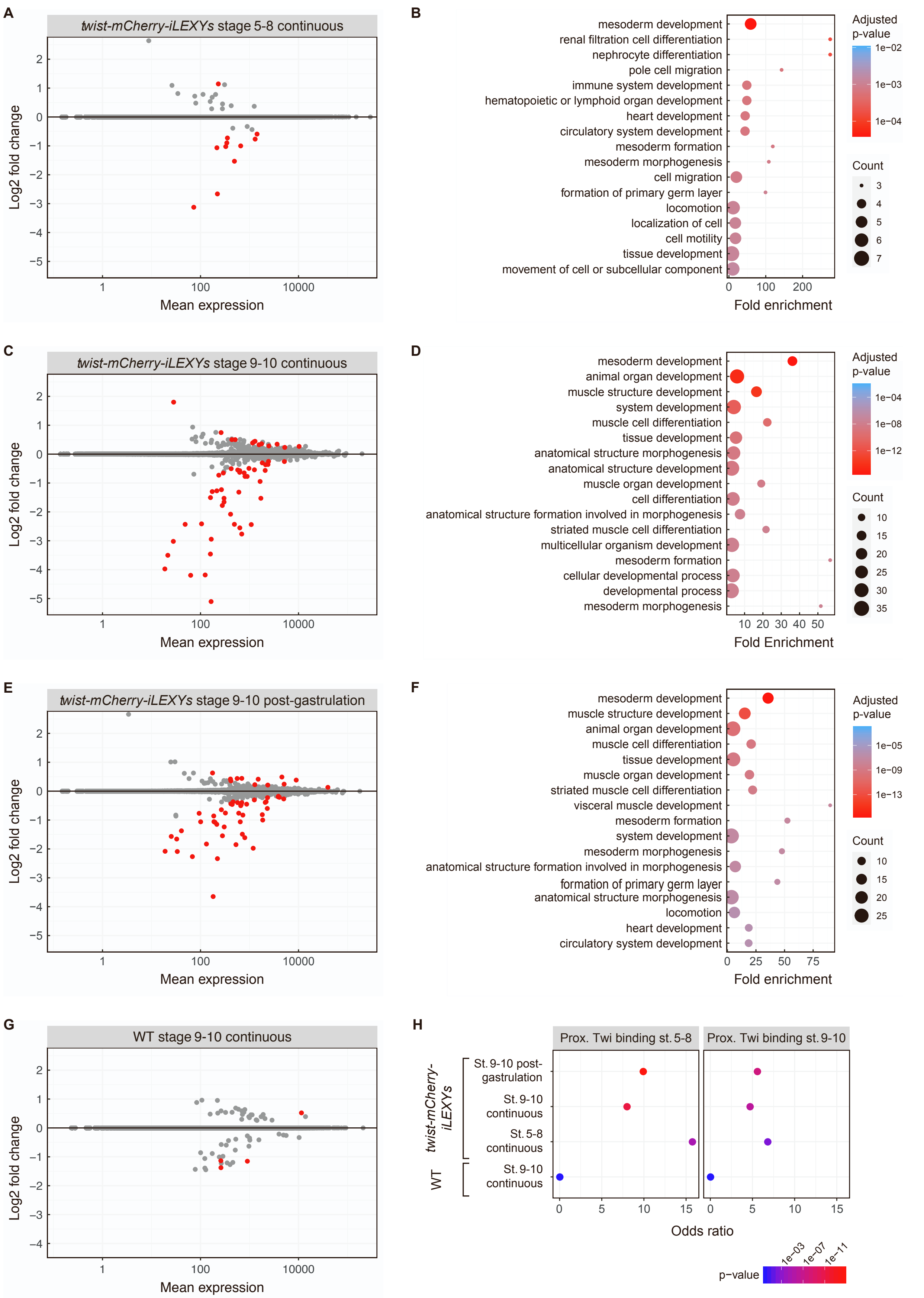
A
twist-mCherry-iLEXYs stage 5-8 continuous
Log2 fold change
Mean expression
B
mesoderm development
renal filtration cell differentiation
nephrocyte differentiation
pole cell migration
immune system development
hematopoietic or lymphoid organ development
heart development
circulatory system development
mesoderm formation
mesoderm morphogenesis
cell migration
formation of primary germ layer
locomotion
localization of cell
cell motility
tissue development
movement of cell or subcellular component
Fold enrichment
Adjusted p-value
Count
C
twist-mCherry-iLEXYs stage 9-10 continuous
Log2 fold change
Mean expression
D
mesoderm development
animal organ development
muscle structure development
system development
muscle cell differentiation
tissue development
anatomical structure morphogenesis
anatomical structure development
muscle organ development
cell differentiation
anatomical structure formation involved in morphogenesis
striated muscle cell differentiation
multicellular organism development
mesoderm formation
cellular developmental process
developmental process
mesoderm morphogenesis
Fold Enrichment
Adjusted p-value
Count
E
twist-mCherry-iLEXYs stage 9-10 post-gastrulation
Log2 fold change
Mean expression
F
mesoderm development
muscle structure development
animal organ development
muscle cell differentiation
tissue development
muscle organ development
striated muscle cell differentiation
visceral muscle development
mesoderm formation
system development
mesoderm morphogenesis
anatomical structure formation involved in morphogenesis
formation of primary germ layer
anatomical structure morphogenesis
locomotion
heart development
circulatory system development
Fold enrichment
Adjusted p-value
Count
G
WT stage 9-10 continuous
Log2 fold change
Mean expression
H
Prox. Twi binding st. 5-8
Prox. Twi binding st. 9-10
twist-mCherry-iLEXYs
St. 9-10 post-gastrulation
St. 9-10 continuous
St. 5-8 continuous
WT
St. 9-10 continuous
Odds ratio
p-value

Figure S5 (related to Figure 6): Genes misexpressed upon nuclear Twist depletion are involved in mesoderm and muscle development

(**A**, **C**, **E**, **G**) MA plots of genes differentially expressed (DE) in stage 5-8 (2-4 h) and stage 9-10 (4-6 h) *twist-mCherry-iLEXYs* or wild-type (WT) embryos upon continuous or post-gastrulation blue light exposure (FDR < 0.01). (**B**, **D**, **F**) Gene ontology (GO) term enrichment for biological process (BP) of DE genes shown in the MA plots on the left. Please note that no genes were DE upon blue light exposure of WT embryos at stage 5-8 and no BP term was found to be significantly enriched in the WT stage 5-8 or stage 9-10 samples. **(H)** Enrichment of DE genes for genes with proximal Twist binding obtained from ChIP data at the same time window of embryogenesis. Dot plots show the odds ratios, colored by significance, of DE genes in the indicated samples and genes closest to stage 5-8 (2-4 h) and stage 9-10 (4-6 h) Twist bound regions (Zinzen et al., 2009).

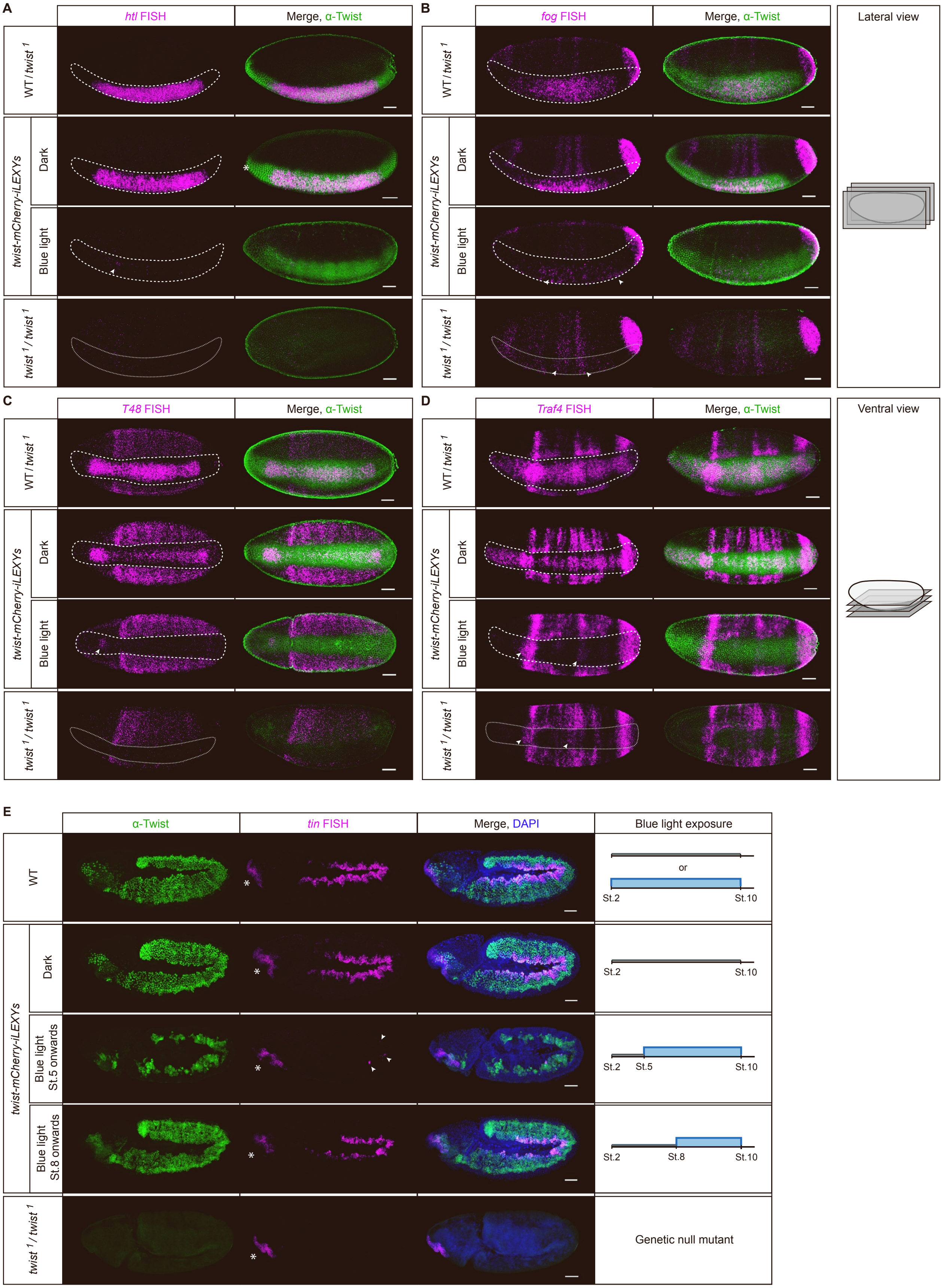

A
htl FISH
Merge, α-Twist
B
fog FISH
Merge, α-Twist
Lateral view
WT / twist 1
twist-mCherry-iLEXYs
Dark
Blue light
twist 1 / twist 1
C
T48 FISH
Merge, α-Twist
D
Traf4 FISH
Merge, α-Twist
Ventral view
E
α-Twist
tin FISH
Merge, DAPI
Blue light exposure
WT
or
St.2
St.10
Blue light St.5 onwards
St.5
Blue light St.8 onwards
St.8
Genetic null mutant

Figure S6 (related to Figure 6): The expression of Twist target genes within the *twist* domain is severely reduced upon nuclear Twist depletion

(A-D) Expression of the characterized Twist target genes *htl* (**A**), *fog* (**B**), *T48* (**C**), and *Traf4* (**D**) at stage 6 visualized by fluorescence *in situ* hybridization (FISH). Maximum intensity projections show blue-light- and dark-incubated *twist-mCherry-iLEXYs* embryos as well as homozygous (*twist*^{1}/*twist*^{1}) and heterozygous (WT/*twist*^{1}) *twist* mutant embryos stained for Twist protein (green) and the mRNA of the indicated genes (magenta) in lateral (A, B) or ventral (C, D) views. Dashed lines indicate the ventral *twist* domain. Target gene expression outside this domain is Twist-independent as indicated by the expression in *twist* loss-of-function (null) mutant embryos (*twist*^{1}/*twist*^{1}). Arrowheads indicate weak residual target gene expression in the Twist expression domain. **(E)** Expression of the Twist target gene *tinman* (*tin*) at stage 10 visualized by FISH. Maximum intensity projections show *twist-mCherry-iLEXYs* embryos incubated in the dark or exposed to blue light from the indicated stage onwards as well as wild-type (WT) and *twist* ^{1}/*twist*^{1} embryos. Embryos were stained for *tin* mRNA (magenta), Twist (green), and DAPI (blue). White asterisks indicate domains where expression is independent of Twist and white arrowheads indicate cells expressing *tin* sporadically. Anterior is left in each panel and dorsal is up in panels A, B, and E. Scale bars represent 50 µm.

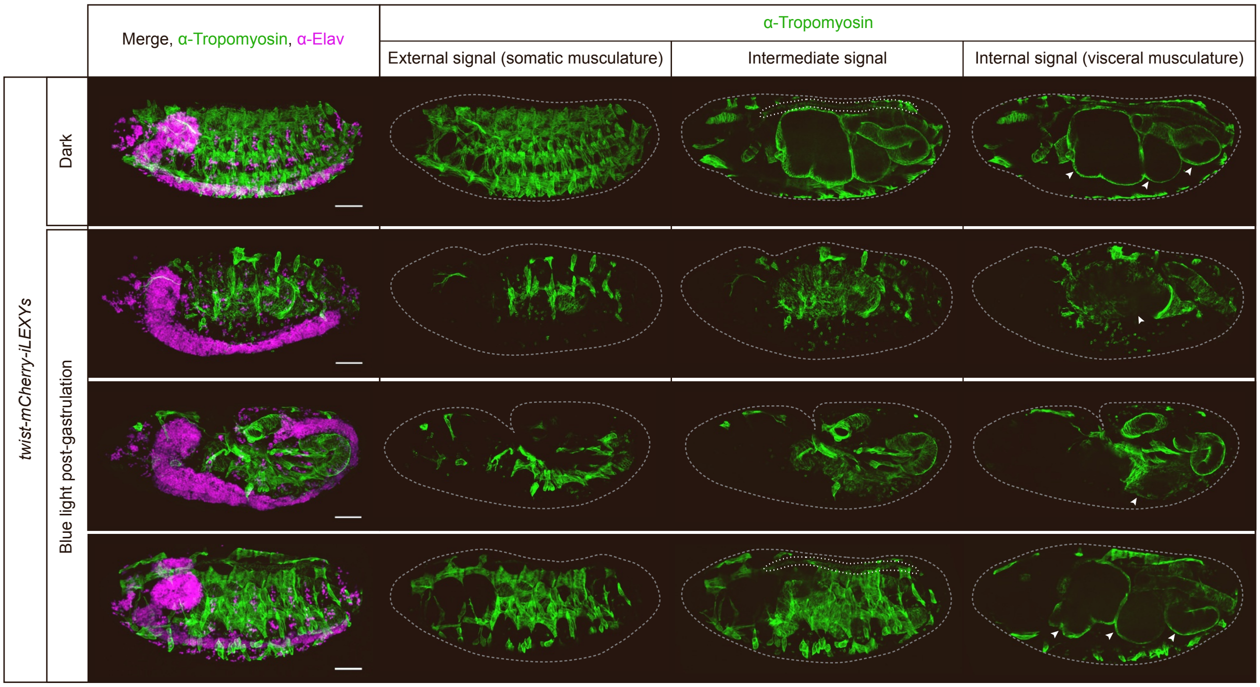

Figure S7 (related to Figure 6): Depletion of nuclear Twist after gastrulation causes defects of different severities in the different muscle tissues

Muscle tissues visualized by α-tropomyosin immunostaining of stage 16 *twist-mCherry-iLEXYs* embryos that were kept in the dark or exposed to blue light only after gastrulation (stage 8 onwards). Maximum intensity projections show images from the external, intermediate, and internal Z-planes of the same embryo within each row. α-tropomyosin (green) was used to stain all muscle types and α-Elav (magenta) was used to stain the nervous system and to facilitate the identification of anatomical locations (leftmost panel). Dashed grey lines indicate the outline of the embryos and dotted white lines mark the cardiac mesoderm in those embryos and Z-planes where it could easily be identified. Arrowheads mark the midgut and different midgut chambers, where applicable. Embryos are shown in lateral views, with dorsal up and anterior left. Scale bars, 50 µm.
